# DR-region of Na^+^/K^+^-ATPase is a target to ameliorate hepatic insulin resistance in obese diabetic mice

**DOI:** 10.7150/thno.46053

**Published:** 2020-05-15

**Authors:** Hai-Jian Sun, Lei Cao, Meng-Yuan Zhu, Zhi-Yuan Wu, Chen-You Shen, Xiao-Wei Nie, Jin-Song Bian

**Affiliations:** 1 Department of Pharmacology, Yong Loo Lin School of Medicine, National University of Singapore, Singapore.; 2 Center of Clinical Research, Wuxi People's Hospital of Nanjing Medical University, Wuxi, Jiangsu 214023, PR China; Lung Transplant Group, Wuxi People's Hospital Affiliated to Nanjing Medical University, Wuxi, Jiangsu 214023, PR China.; 3 National University of Singapore (Suzhou) Research Institute, Suzhou, China.

**Keywords:** diabetes, insulin resistance, Na^+^/K^+^-ATPase, gluconeogenesis, glycogenesis

## Abstract

Reduced hepatic Na^+^/K^+^-ATPase (NKA) activity and NKAα1 expression are engaged in the pathologies of metabolism diseases. The present study was designed to investigate the potential roles of NKAα1 in hepatic gluconeogenesis and glycogenesis in both hepatocytes and obese diabetic mice.

**Methods**: Insulin resistance was mimicked by glucosamine (GlcN) in either human hepatocellular carcinoma (HepG2) cells or primary mouse primary hepatocytes. Obese diabetic mice were induced by high-fat diet (HFD) feeding for 12 weeks.

**Results**: We found that both NKA activity and NKAα1 protein level were downregulated in GlcN-treated hepatocytes and in the livers of obese diabetic mice. Pharmacological inhibition of NKA with ouabain worsened, while activation of NKAα1 with an antibody against an extracellular DR region of NKAα1 subunit (DR-Ab) prevented GlcN-induced increase in gluconeogenesis and decrease in glycogenesis. Likewise, the above results were also corroborated by the opposite effects of genetic knockout/overexpression of NKAα1 on both gluconeogenesis and glycogenesis. In obese diabetic mice, hepatic activation or overexpression of NKAα1 stimulated the PI3K/Akt pathway to suppress hyperglycemia and improve insulin resistance. More importantly, loss of NKA activities in NKAα1^+/-^ mice was associated with more susceptibility to insulin resistance following HFD feeding.

**Conclusions**: Our findings suggest that NKAα1 is a physiological regulator of glucose homoeostasis and its DR-region is a novel target to treat hepatic insulin resistance.

## Introduction

It is well known that obesity and insulin resistance are the driving forces for the development of various comorbidities, such as diabetes, cardiovascular diseases, nonalcoholic fatty liver disease, and even several types of cancers 1, 2. Over the past decades, studies have shown that diet-induced obesity promotes insulin resistance by complex mechanisms that involve genetic background, hyperinsulinemia, increased amount of non-esterified fatty acid (NEFA) and glycerol, mitochondrial dysfunction, oxidative stress, endoplasmic reticulum stress, inflammation response, gut microbiota and other factors 3-5. However, none of those hypotheses has led to effective therapies for obesity and insulin resistance. The main reason is that the underlying mechanisms of obesity-related insulin resistance have yet to be fully elucidated. As such, a better understanding of molecular mechanism or identification of novel targets for insulin resistance in obesity may facilitate the development of promising strategies to eradicate or reduce adverse metabolic consequences of obesity 6-8.

In addition to systemic insulin resistance, hepatic insulin resistance is also a core event in the pathogenesis of metabolic syndrome, obesity, and type 2 diabetes 9, 10. The liver is the first organ where insulin reaches after being secreted from the pancreas and it plays an indispensable role in glucose storage and disposal through the regulation of gluconeogenesis, glycogenesis and glycogenolysis 11. Gluconeogenesis is an important pathway for glucose production, whereas glycogenesis is a player in glucose utilization/storage 12. Gluconeogenesis is upregulated in response to hormones during fasting process 13, which is primarily regulated by phosphoenolpyruvate carboxykinase (PEPCK) and glucose-6-phosphatase (G6pase) 14. The elevated hepatic gluconeogenesis is detectable in both obesity and type 2 diabetes 15, 16. Glycogen synthesis (glycogenesis) is mainly modulated by glycogen synthase kinase-3 (GSK3) and glycogen synthase (GS) 12. Dysfunction of GSK3 or GS results in a decrease in fluxes of glycogenesis, thereby leading to excessive glucose generation and hyperglycemia 17. Excessive hepatic glucose production is involved in fasting hyperglycemia and aggravated postprandial hyperglycemia in both types 1 and 2 diabetes 18. Therefore, modulating either activities or gene expressions of these metabolic enzymes that participate in hepatic gluconeogenesis and glycogenesis may be beneficial for the correction of insulin resistance and hyperglycemia.

Na^+^/K^+^ ATPase (NKA) is a transmembrane protein that exerts an important role in the regulation of cellular functions 19. Structurally, NKA is mainly composed of three subunits α, β, and γ 20. Decreased NKA activity and its altered isoform expression might contribute to cardiac dysfunction, myocardial dilation and heart failure 21-23. Also, it is established that diabetic cardiomyopathy is closely related to advanced glycation end-products (AGE)-induced NKA activity impairment 24. Obesity is associated with hyperglycemia and hyperinsulinemia, which may suppress or inactivate the enzyme of NKA 25. When compared with control mice, the NKA activity in the liver is reduced by 63% in hyperglycemic-hyperinsulinemic ob/ob mice 26. In similarity, the activity of NKA is also reduced in adipose tissue from obese patients in comparison with lean subjects 27. In diabetic rats, the activity of NKA is also declined in the livers, while the antidiabetic compounds could restore the downregulated NKA activity in diabetic liver tissues 28. Moreover, the decreased NKA activity and NKAα1 protein expressions are observed in the livers from high-fat diet (HFD) rats 29. Ouabain, a classic drug known to inhibit NKA activity and function, is found to increase the synthesis of cholesterol in human HepG2 cells 30. Very recently, it is reported that NEFA may induce the accumulation of adenine nucleotide metabolite, thereby inhibiting hepatic NKA activity in type 2 diabetic mice 31. It is, therefore, highly probable that NKAα1 is a critical gene that participates in glycolipid metabolism homeostasis. However, the roles and precise mechanisms of NKAα1 in obesity and its related insulin resistance remain uncharacterized. In this study, we aimed to explore the impacts of NKAα1 on gluconeogenesis and glycogenesis in both hepatocytes and HFD-induced obese diabetic mice and to elucidate the potential mechanisms.

## Material and Methods

### Reagents

Dulbecco's Modified Eagle's Medium (DMEM), trypsin-EDTA, penicillin-streptomycin solution, and fetal bovine serum (FBS) were acquired from Hyclone (South Logan, UT, USA). Antibodies against NKAα1, NKAα2, NKAα3, P110α, P110β, PEPCK, G6pase, β-actin, β-tubulin, GAPDH, and the secondary antibodies were purchased from Santa Cruz Biotechnology (Santa Cruz, CA, USA). Alexa Fluor 568 conjugated goat anti-rat IgG (H+L) was purchased from Invitrogen Corporation (Carlsbad, USA). Antibodies against pan-cadherin, phospho-GS (Ser641), phospho-GSK3 (Ser9), GS and GSK3 were obtained from Abcam (Cambridge, MA, USA). The generation of a DR-region-specific antibody (^897^DVEDSYGQQWTYEQR^911^) was performed as our previous reports 32, 33. Hematoxylin and eosin (H&E) staining kit, G6pase and PEPCK activity kits, periodic acid-schiff (PAS) kit, glucose assay kit, insulin assay kit, and glycogen assay kit were obtained from Nanjing Jiancheng Bioengineering Institute (Nanjing, China). The specific primers were provided by Integrated DNA Technologies Pte. Ltd. (Singapore). LY294002 and MK2206 were bought from Selleck Chemicals (Houston, TX, USA). D12492 60 kcal% fat was obtained from Research Diets (New Brunswick, NJ, USA). Glucosamine (GlcN), insulin, glucose, and phosphate colorimetric kit were obtained from Sigma (St. Louis, USA).

### Animals

All animal experimental procedures in the present study were approved and monitored by the Institutional Animal Care and Use Committee of the National University of Singapore and Wuxi People's Hospital Affiliated to Nanjing Medical University. All animal experiments were carried out according to the relevant guidelines. Mice were housed in a temperature-controlled and humidity-controlled room with food and tap water ad libitum. NKAα1^+/-^ mice were kindly provided by Dr. Jerry B. Lingrel in the University of Cincinnati, USA 34. Male wild-type (WT) C57/BL6J mice or NKAα1^+/-^ mice aged 5-6 weeks were subject to high fat diet (HFD, 60% kcal as fat) for 12 weeks to induce obese diabetes as previous reports 35. Mice in the control group were fed with a normal chow diet (Ctrl, 12% kcal as fat). For lentivirus-mediated NKAα1 overexpression experiment, after feeding with a normal diet or HFD diet for 8 weeks, a single intravenous injection of lentivirus (20 μl stock dissolved in 100 μl PBS) expressing NKAα1 or control vector was carried out according to manufacturer's protocols. Two weeks later, a repeat injection of such lentiviral activation particles via tail vein was conducted to ensure overexpression of NKAα1. Acute experiments were performed 4 weeks after the first introduction of NKAα1. To examine whether DR-Ab attenuated hyperglycemia and improved insulin resistance, WT C57BL/6J mice were fed by control diet or HFD for 6 weeks, and the mice were then subject to intraperitoneal injection of normal IgG or DR-Ab (2 mg/kg/every other day) for next 6 weeks 36. At the end of experiments, the blood glucose of mouse tail vein was detected by the Roche Accu-Chek monitor. After that, the blood was collected by cardiac puncture to measure insulin levels using commercial kits, and the livers were harvested for gene expression and histological analysis.

### Cell culture and treatments

HepG2 cells (American Type Culture Collection, Manassas, VA, USA) were cultured in DMEM supplement with 10% FBS, penicillin (100 U/ml) and streptomycin (100 μg/ml) in a humidified incubator containing 5% CO_2_ at 37 °C. GlcN is well established to induce insulin resistance and glucose intolerance in a number of tissues, including the muscle, liver and adipose through activation of the hexosamine biosynthetic pathway 37-41. In parallel with this, incubation of hepatocytes with high amount of GlcN is effective to mimic insulin-resistant hepatic cell model via increased hexosamine biosynthetic pathway 42, 43. In order to induce insulin resistance *in vitro*, HepG2 cells were stimulated by GlcN (18 mM) for 18 h, followed by incubation with ouabain (10 nM), DR-Ab (2 μM) or vehicle for 30 min for measurement of phosphorylated protein, and 24 h for other measurements. The phosphoinositide 3-kinase (PI3K) inhibitor LY294002 (10 μM) and the Akt inhibitor MK2206 (1 μM) were pre-added into the medium 24 h before the induction of insulin resistance model in HepG2 cells. Primary hepatocytes were collected from male WT C57BL/6J mice and NKAα1^+/-^ mice by using collagenase II (0.66 mg/ml) at 37 °C as previously described 44. The primary mouse hepatocytes were maintained in DMEM containing 10% FBS, penicillin (100 U/ml) and streptomycin (100 μg/ml) for 24 h before inducing insulin resistance by GlcN.

### Generation of stable HepG2 cell with NKAα1 knockout or overexpression

The NKAα1 CRISPR/Cas9 KO Plasmids (h) and NKAα1 HDR Plasmid (h) were ordered from Santa Cruz Biotechnology (Santa Cruz, CA, USA). HepG2 cells were transfected with these plasmids and then selected with puromycin (5 µg/ml). After selection, the clones were picked up and identified with western blot. After identification the NKA knockout stable cell line was used for the following experiments. For the stable overexpression of NKAα1 in HepG2 cells, the plasmid of SNAP-HA-NKAα1 was a kind of gift from Dr. Michael Caplan (Yale University School of Medicine). SNAP-HA-NKAα1 was transfected to cells and then selected with 800 µg/ml of G418. In addition, during the initial selection process, ouabain (10 µM) was also added for selecting clones displaying active NKA at the cell surface. After selection, the clones were picked up and identified with western blot. After identification, the NKAα1 overexpression stable cell line was used for the following experiments.

### Assessment of glucose and glycogen levels

The measurement of glucose and glycogen content was performed using commercial kits according to the manufacturer's recommendations. In brief, the cell culture medium was exchanged with DMEM supplemented with sodium pyruvate (2 mM) and sodium lactate (20 mM) in the absence of phenol red. After incubation for 3 h, the glucose generation buffer was collected and determined by a glucose oxidase-peroxidase assay kit (Jiancheng Bioengineering Institute, Nanjing, China). The glycogen can be dehydrated to form an aldaldehyde derivative under the action of concentrated sulfuric acid, and the latter reacted with the anthrone to form a blue compound. Based on this, glycogen levels were determined with the aid of a glycogen assay kit (Jiancheng Bioengineering Institute, Nanjing, China) following the manufacturer's protocols. Finally, the data were normalized to the total protein levels in each sample. In addition, to observe the glycogen store in HepG2 cells, the collected cells fixed with 4% paraformaldehyde and then subject to PAS staining, the red staining parts is visualized as glycogen under light Olympus BX50 microscopy.

### Measurement of PEPCK and G6pase activities

The activities of G6pase and PEPCK were examined by commercially available kits (Nanjing Jiancheng Bioengineering Institute, Nanjing, China). In short, reaction of PEPCK with oxaloacetate produces phosphoenolpyruvate and carbon dioxide, which was then catalyzed to the formation of NAD+ from NADH, by pyruvate kinase and lactate dehydrogenase. This reaction was monitored by a decrease in NADH at 340 nm. G6pase activity was detected by quantifying the formation of NADPH from NADP at 340 nm. The activities of G6pase and PEPCK were then normalized to the protein content in each sample.

### Glucose tolerance test (GTT) and insulin tolerance test (ITT)

GTT and ITT were measured to evaluate insulin resistance and glucose tolerance as previously described 45, 46. In brief, to assess insulin sensitivity and glucose tolerance, mice were fasted for 6 hours and overnight, respectively. The mice were then received intraperitoneal injection of insulin (0.75 units/kg body weight) or glucose (2.0 g/kg body weight), respectively. After that, the blood glucose levels were tested by blood glucose monitor at 0, 15, 30, 60 and 120 min after injection of insulin or glucose.

### Histological analysis

After perfusion with PBS, the liver tissues were incubated in 4% paraformaldehyde, embedded in paraffin, and sectioned (5-μm thickness), followed by staining with PAS to visualize the glycogen deposits in livers. The liver sections were oxidized in periodic acid for 20 min in the dark and washed by deionized water for three times. After incubation with 0.5% sodium bisulfite in 0.05 M HCl for 10 min, sections were counterstained with haematoxylin, and the liver sections were captured by using light Olympus BX50 microscopy. In addition, immunofluorescence was carried out to detect the NKAα1 protein expression in the livers from mice.

### Cell surface labeling and immunoblot analysis

To isolate the cell plasma membrane, cells were labeled by EZ-link Sulfo-NHSSS-biotin (1 mg/ml, Pierce, USA) for 1 h and pulled down with streptavidin as previously described 47. Equal contents of total protein were separated onto sodium dodecyl sulfate-polyacrylamide gel electrophoresis (SDS-PAGE) and transferred to PVDF membrane. The membranes were incubated with primary antibodies and required secondary horseradish peroxidase-conjugated antibodies, and then developed. The targeted protein expressions were normalized to the internal reference gene on the same membrane. The plasma membrane protein of NKAα1 was normalized to pan-cadherin.

### Quantitative real-time PCR

Real-time PCR was carried out by using a VIIA(TM) 7 System (Applied Biosystems, Foster City, CA, USA). The gene expressions were calculated by normalization against GAPDH. The sequences of primers used are listed in **Table S1** and **Table S2**).

### Antibody generation and purification

The detailed methods for the generation and purification of DR-Ab were performed in our previous reports 32, 33. In short, the rats were subject to keyhole limpet hemocyanin (KLH) conjugated DR peptide (897-DVEDSYGQQWTYEQR-911) subcutaneously every two weeks with an initial dose of 200 μg protein emulsified with complete Freund's adjuvant (CFA) followed by 100 μg protein emulsified with incomplete Freund's adjuvant for three times. The serum of immunized rats was collected and purified with protein A/G spin column (Thermo Fisher Scientific, 89962) on the basis of the manufacturer's procedures.

### NKA activity measurement

The NKA activity was measured based on the previous protocols 48. The samples were homogenized in buffer A (20 mM HEPES, 250 mM sucrose, 2 mM EDTA, 1 mM MgCl_2_, pH 7.4). The protein levels were analyzed by Bradford colorimetric protein assay kit (Rockford, IL. USA). Two 50 μl aliquots of homogenate were collected, which is incubated with the presence or absence of ouabain (2 mM), and the reaction was initiated in the presence of ATP (1 mM). Reactions were stopped by 10 μL of 100% (w/v) trichloroacetic acid. Samples were incubated on ice for 1 h and then centrifuged to pellet the precipitated protein. The phosphate levels were detected by spectrophotometric assay kit (Sigma, St. Louis, MO, USA) at the absorbance of 650 nm. Enzyme specific activities were calculated from the inorganic phosphorous production from the decomposition per mg protein per hour.

### Statistics

The results were presented as Mean ± SEM and the statistical analysis was implemented by using the Statistical Program for Social Sciences (SPSS, version 17.0). ANOVA was applied for comparisons among multiple group comparisons. Post-hoc comparisons were achieved by Bonferroni's multiple comparisons test depending on the experiments. Student's unpaired two-tailed* t* test was employed when two groups were compared. P < 0.05 was thought to have statistical significance.

## Results

### Biogenesis of NKAα1 was impaired in HepG2 hepatocytes with insulin resistance and in the livers of obese diabetic mice

**Figure 1:**
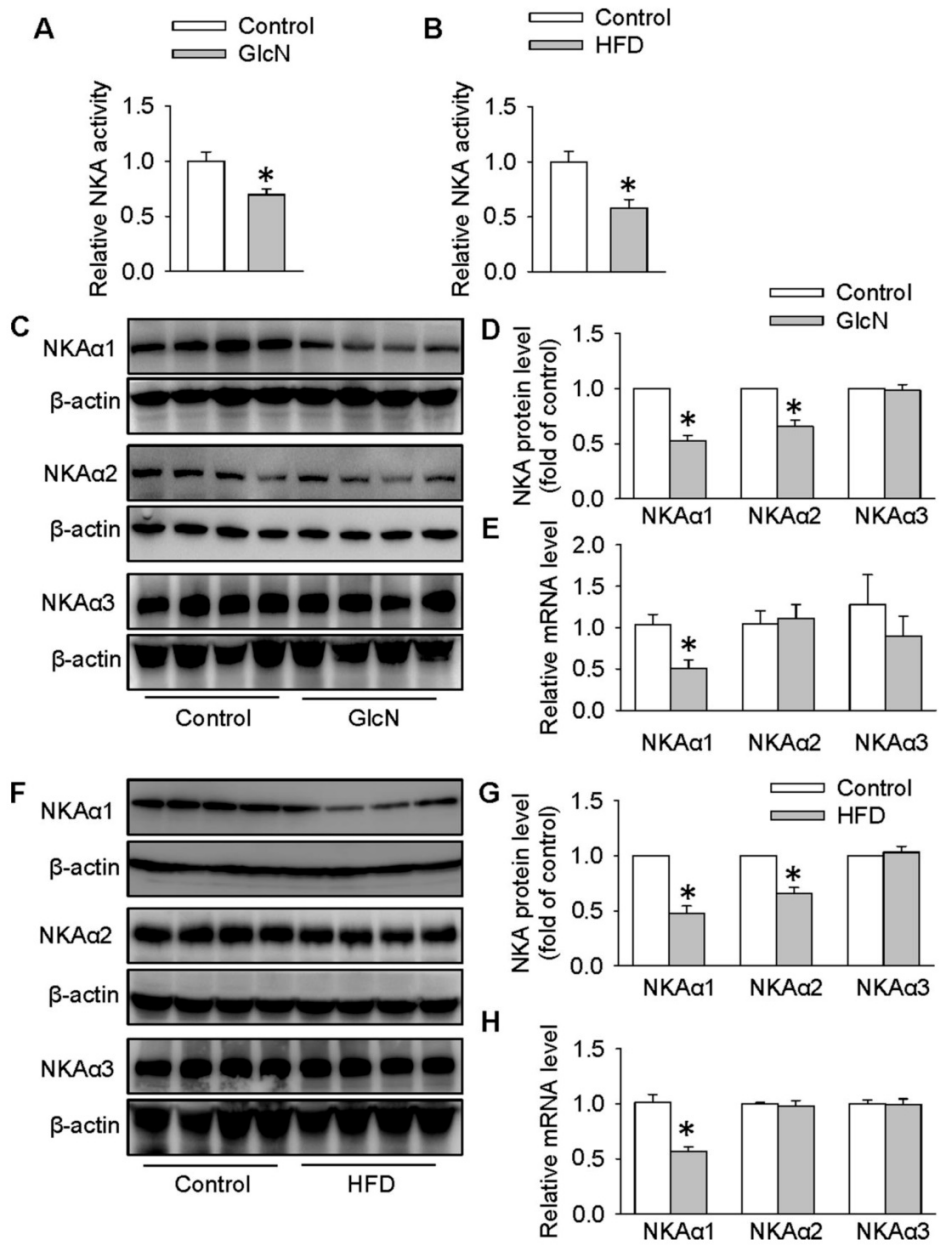
** Biogenesis of NKAα1 was impaired in HepG2 hepatocytes with insulin resistance and livers of obese diabetic mice**. (**A**) NKA activity was decreased in GlcN-treated HepG2 cells. (**B**) NKA activity was inhibited in the livers from HFD mice. (**C, D**) Representative immunoblots and quantification analysis of the protein expressions of NKAα1, NKAα2 and NKAα3 in HepG2 cells treated with GlcN (18 mM) for 18 h in DMEM with 5 mM glucose. (**E**) Relative mRNA levels of NKAα1, NKAα2 and NKAα3 in HepG2 cells. (**F, G**) Representative immunoblots and quantification analysis of the protein expressions of NKAα1, NKAα2 and NKAα3 in the livers from control mice and HFD mice. (**H**) Relative mRNA levels of NKAα1, NKAα2 and NKAα3 in the livers. Data were expressed as Mean ± SEM. * P < 0.05 vs. Control. The results were calculated from 4 to 8 independent experiments.

In the presence of high concentration of GlcN, the actions of insulin and subsequent insulin signaling pathways are dramatically blocked, resulting in glucose metabolism misalignment in hepatocytes 44, 49. In HepG2 cells, GlcN increases gluconeogenesis and decreases glycogen synthesis which is similar with hepatic insulin resistance in animals 50. For this reason, GlcN was applied to induce insulin resistance in HepG2 cells or primary hepatocytes. To determine whether NKA was involved in hepatic insulin resistance, we measured the NKA activity and the protein expressions of its various isoforms in both HepG2 hepatocytes treated with GlcN and in the livers from obese diabetic mice. As shown in **Figure 1**, significant reductions in NKA activity were found in both hepatocytes with insulin resistance (**Figure 1A**) and livers from obese diabetic mice (**Figure 1B**). To study the involved NKA isoforms, we examined the protein expressions of NKA isoforms in above cells or tissues. As shown in **Figure 1C-E**, GlcN incubation markedly reduced the expression of NKAα1 at both protein (**Figure 1C-D**) and mRNA (**Figure 1E**) levels in HepG2 hepatocytes. The protein expressions of NKAα2 were also downregulated in GlcN-treated HepG2 hepatocytes. However, no significant change was found in its mRNA expression, suggesting that the effect of GlcN on NKAα2 was at post-transcriptional level. We also failed to see significant changes in NKAα3 at both protein and mRNA levels between control cells and GlcN-incubated HepG2 hepatocytes (**Figure 1C-E**). Similar results were also observed in the livers of obese diabetic mice (**Figure 1F-H**). Taken together, our data suggest that hepatic NKAα1 may be an important protein to be affected during obese diabetic disease.

### NKAα1 inhibition/knockout exacerbated gluconeogenesis and glucose production in hepatocytes

**Figure 2:**
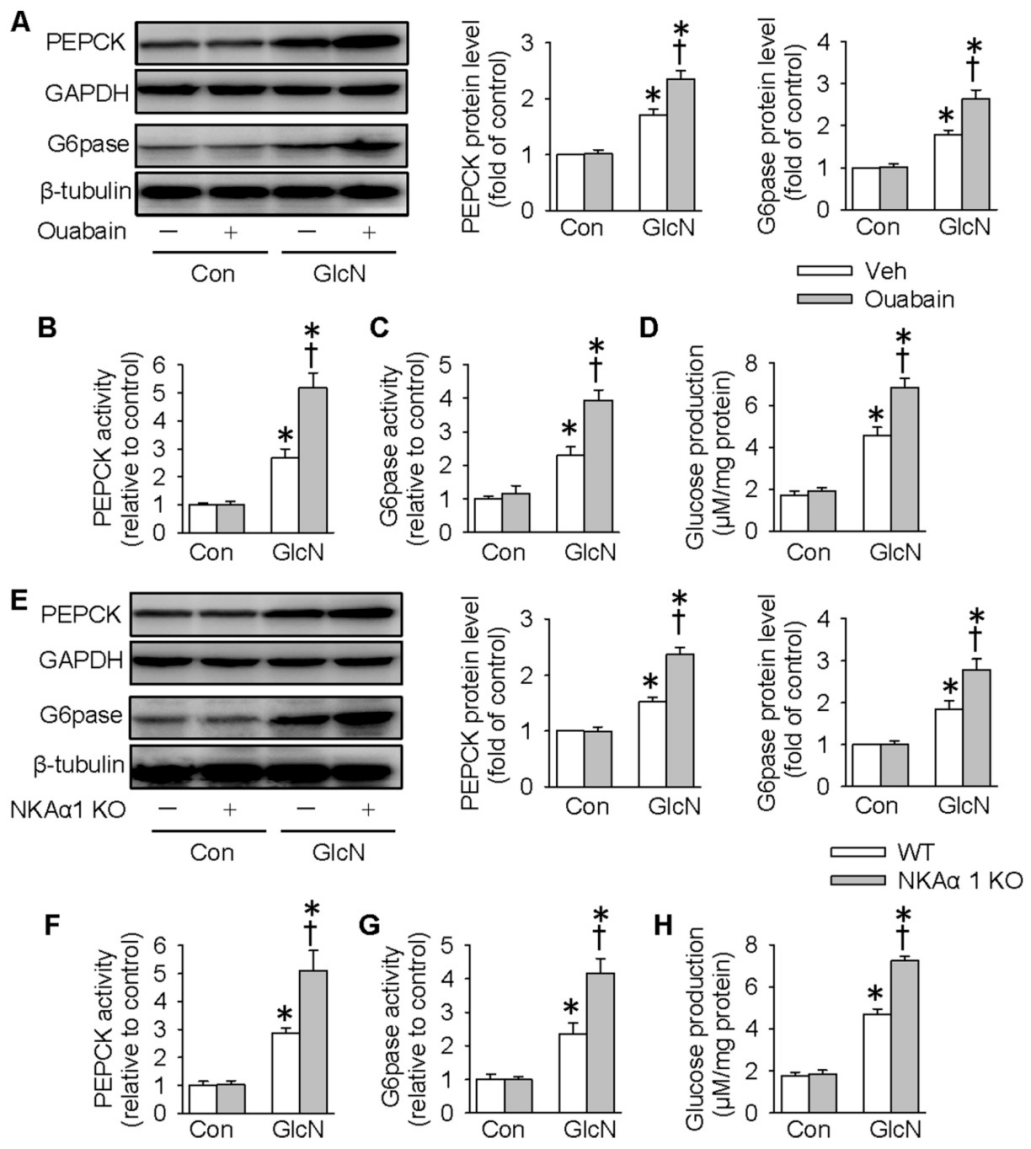
** NKAα1 inhibition/knockout exacerbated gluconeogenesis and glucose production in hepatocytes.** Ouabain, an NKAα1 inhibitor, further deteriorated the effects of GlcN on the protein expressions of PEPCK and G6pase (**A**), PEPCK and G6pase activities (**B, C**) as well as glucose production (**D**). NKAα1 deficiency further aggravated the actions of GlcN on the protein expressions of PEPCK and G6pase (**E**), PEPCK and G6pase activities (**F, G**) as well as glucose production (**H**). Data were expressed as Mean ± SEM. * P < 0.05 vs. Control (Con). † P < 0.05 vs. Vehicle (Veh) or wild-type (WT). The results were calculated from 4 to 6 independent experiments.

Excessive gluconeogenesis is a critical player in hepatic insulin resistance and impaired glucose metabolism 51. We next determined whether NKAα1 is involved in hepatic gluconeogenesis by determining the expressions and activities of PEPCK and G6pase, two important enzymes that participate in the process of hepatic gluconeogenesis 14. Consistent with previous studies 44, GlcN treatment upregulated the protein expressions and activities of PEPCK and G6pase, as well as glucose production in HepG2 cells (**Figure 2A-H**). Pharmacological inhibition of NKA with ouabain further enhanced the above stimulatory effects of GlcN (**Figure 2A-D**). To confirm the effect of endogenous NKA, we deleted NKAα1 expression with the CRISPR/Cas9 technique (**Figure S1A**). Although NKAα1 gene ablation did not affect the basal hepatic gluconeogenesis, it also enhanced the effect of GlcN on both protein expressions (**Figure 2E**) and activities (**Figure 2F-G**) of PEPCK and G6pase and glucose production (**Figure 2H**). We also tested the effect of GlcN in the primary cultured hepatocytes isolated from WT and NKAα1^+/-^ mice. The loss of NKA in NKAα1^+/-^ mice was verified in**Figure S2A**. As shown in **Figure S2**, NKAα1 loss further enhanced the actions of GlcN on the protein expressions of PEPCK (**Figure S2B**) and G6pase (**Figure S2C**). Taken together, the above data suggest that either NKA inhibition or NKAα1 deficiency rendered the HepG2 hepatocytes more vulnerable to insulin resistance.

### NKAα1 activation/overexpression abrogated gluconeogenesis and glucose production in hepatocytes

**Figure 3:**
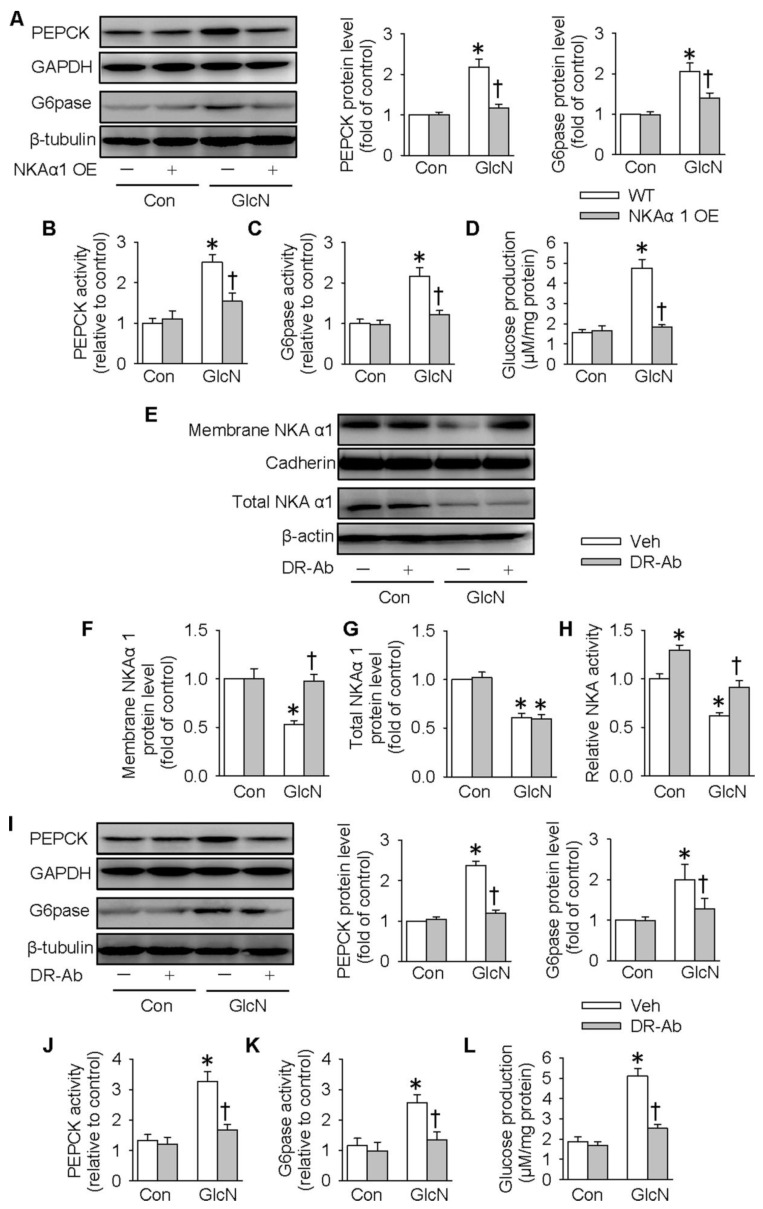
** NKAα1 activation/overexpression exacerbated gluconeogenesis and glucose production in hepatocytes.** NKAα1 overexpression attenuated the actions of GlcN on the protein expressions of PEPCK and G6pase (**A**), PEPCK and G6pase activities (**B, C**) as well as glucose production (**D**). (**E-G**) DR-Ab treatment reversed GlcN-induced loss of plasma membrane NKA α1, but had no effect on the total NKA α1 protein expression. (**H**) DR-Ab treatment reversed GlcN-induced downregulation of NKA activity. NKAα1 activator DR-Ab reversed the effects of GlcN on the protein expressions of PEPCK and G6pase (**I**), PEPCK and G6pase activities (**J, K**) as well as glucose production (**L**). Data were expressed as Mean ± SEM. * P < 0.05 vs. Control (Con). † P < 0.05 vs. Vehicle (Veh) or wild-type (WT). The results were calculated from 4 to 6 independent experiments.

We next determined whether overexpression of NKAα1 can attenuate the effects of GlcN on insulin resistance. As shown in **Figure 3A-D**, overexpression of NKAα1 (**Figure S1B**) significantly reduced the stimulatory effects of GlcN on the protein expressions (**Figure 3A**) and activities (**Figure 3B-C**) of PEPCK and G6pase as well as the glucose formation (**Figure 3D**). We developed an antibody against specific DR-region of NKAα1 (DR-Ab) which stimulates NKA activity and preserves membrane NKAα1 expressions 52, 53. We found that in the present study it also reversed GlcN-induced membrane loss of NKAα1 (**Figure 3E-F**) and decreased NKA activity (**Figure 3H**), although it had no significant effect on total NKAα1 protein expression (**Figure 3E and 3G**). Similarly, we found that DR-Ab also attenuated the effects of GlcN on gluconeogenesis (**Figure 3I-L**). To sum up, our data indicate that activation or stabilization of membrane NKAα1 might be potential approaches for inhibition of gluconeogenesis under insulin resistance conditions.

### Role of NKAα1 in glycogen synthesis and glycogen content in hepatocytes

**Figure 4:**
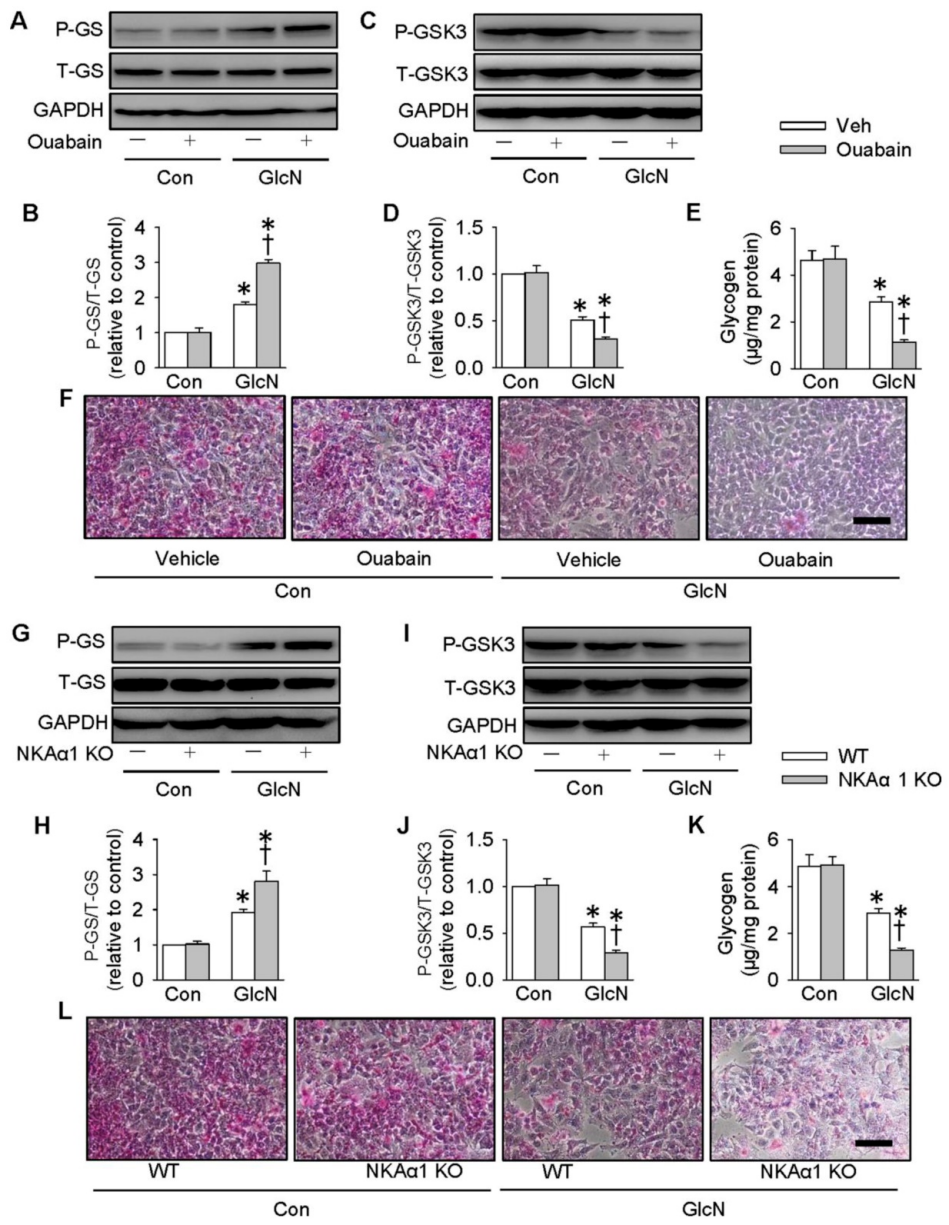
** Role of NKAα1 inhibition/knockout in glycogen content in hepatocytes.** Inhibition of NKAα1 by ouabain further decreased glycogen synthesis in hepatocytes challenged by GlcN, as evidenced by measurement of GS phosphorylation (**A, B**), GSK3 phosphorylation (**C, D**), glycogen content (**E**) and PAS staining (**F**). Likewise, knockout of NKAα1 exacerbated the effects of GlcN on GS phosphorylation (**G, H**), GSK3 phosphorylation (**I, J**), glycogen content (**K, L**) in HepG2 cells. Data were expressed as Mean ± SEM. * P < 0.05 vs. Control (Con). † P < 0.05 vs. Vehicle (Veh) or wild-type (WT). The results were calculated from 4 to 6 independent experiments.

It is reported that ectopic lipid accumulation in the liver triggers insulin resistance and this effect is dependent on decreased hepatic glycogen synthesis 54. Inhibition of GSK3 and de-phosphorylation of GS are considered as promising strategies for restoring hepatic glycogen synthesis during insulin resistance 55. It was found that GlcN treatment upregulated GS phosphorylation (**Figure 4A-B**), but downregulated GSK3 phosphorylation (**Figure 4C-D**) and glycogen content (**Figure 4E**) in HepG2 hepatocytes. These effects were further aggravated by inhibition of NKA with ouabain. PAS staining further confirm that the glycogen synthesis was lower in GlcN-treated HepG2 hepatocytes and this was worsened when treated with ouabain (**Figure 4F**). Similar to the effect of ouabain, gene deletion of NKAα1 also enhanced the effects of GlcN on the phosphorylation of GS (**Figure 4G-H**), phosphorrylation of GSK3 (**Figure 4I-J**), glycogen content (**Figure 4K**) and synthesis (**Figure 4L**) in HepG2 hepatocytes. The effects of NKAα1 loss on phosphorylation of GS (**Figure S2D**) and GSK3 (**Figure S2E**) were also confirmed in GlcN-treated mouse primary hepatocytes isolated from both NKAα1^+/+^ and NKAα1^+/-^ mice.

**Figure 5:**
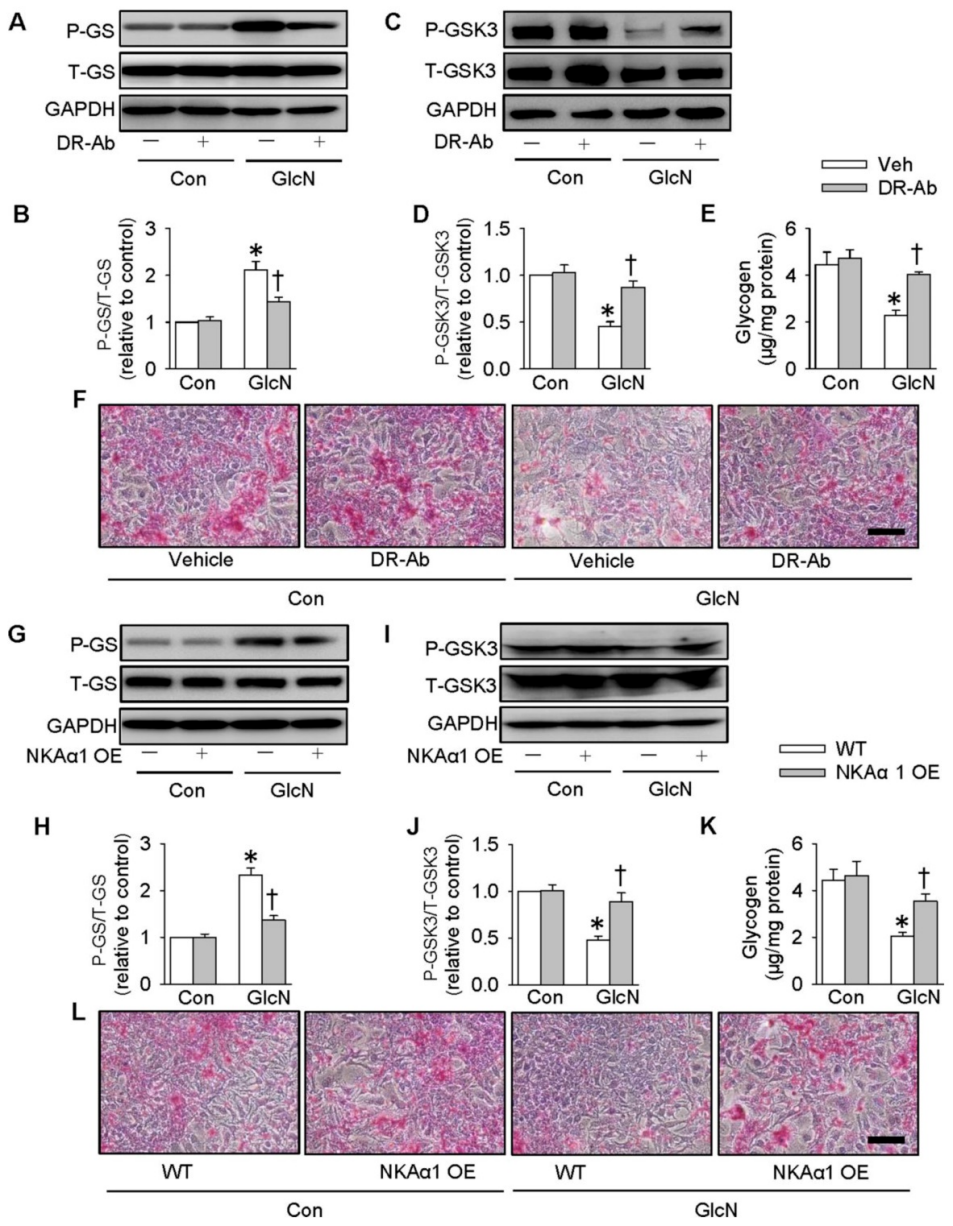
** Role of NKAα1 activation/overexpression in glycogen synthesis in hepatocytes.** Activation of NKAα1 by DR-Ab prevented the reduced glycogen content in hepatocytes challenged by GlcN, as evidenced by measurement of GS phosphorylation (**A, B**), GSK3 phosphorylation (**C, D**), glycogen content (**E**) and PAS staining (**F**). Similarly, overexpression of NKAα1 antagonized the effects of GlcN on GS phosphorylation (**G, H**), GSK3 phosphorylation (**I, J**), glycogen content (**K, L**) in HepG2 cells. Data were expressed as Mean ± SEM. * P < 0.05 vs. Control (Con). † P < 0.05 vs. Vehicle (Veh) or wild-type (WT). The results were calculated from 4 to 6 independent experiments.

On the contrary, DR-Ab-mediated NKAα1 activation (**Figure 5A-F**) and overexpression of NKAα1 (**Figure 5G-L**) were capable of reversing the effects of GlcN on the phosphorylation of GS and GSK3, as well as glycogen content and synthesis. These above findings suggested that NKAα1 inhibition/knockout exacerbated, while NKAα1 activation/overexpression dampened the decreased glycogen synthesis in hepatocytes with insulin resistance.

### NKAα1 regulated hepatic glucose metabolism through the PI3K/Akt pathway

**Figure 6:**
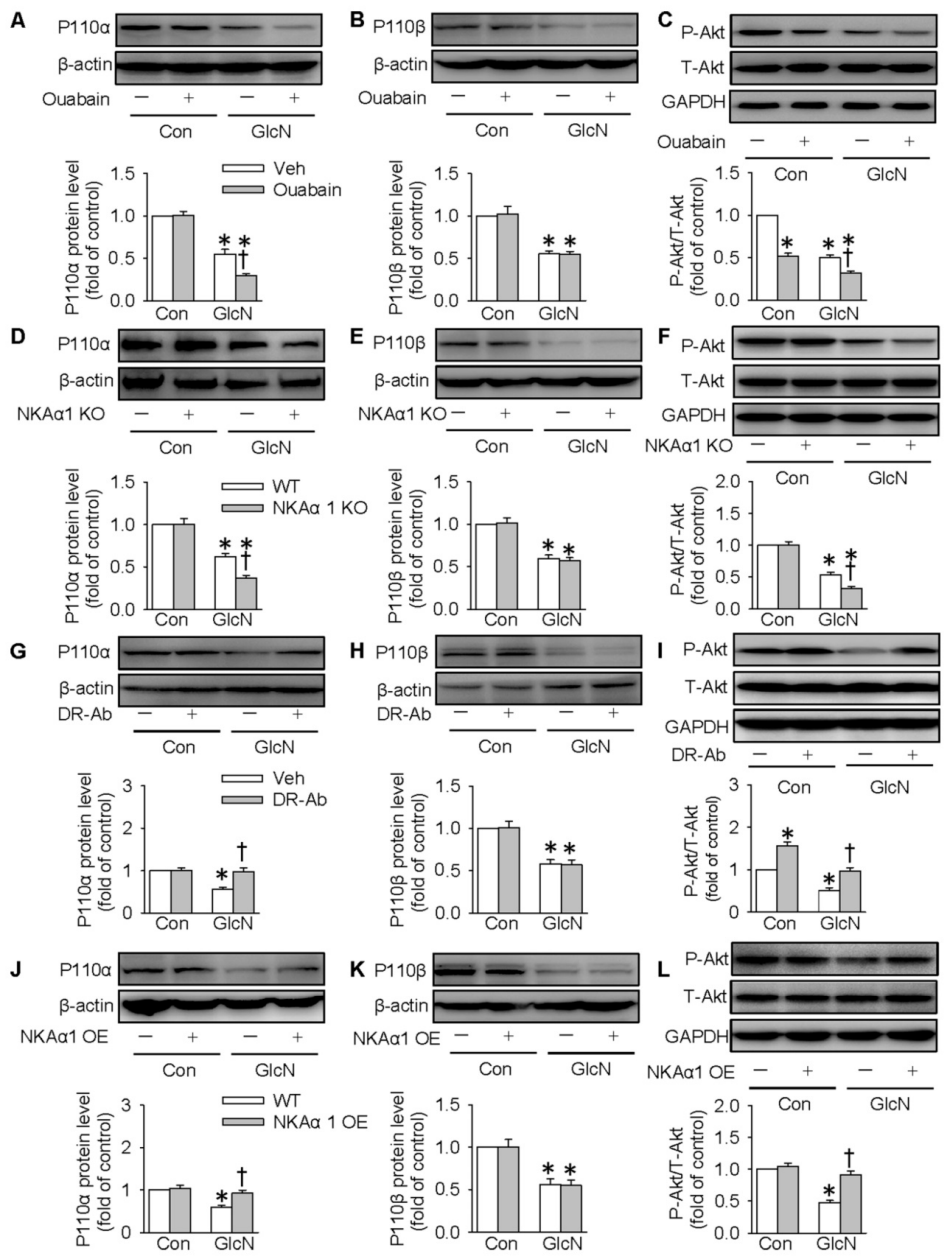
** Role of NKAα1 in the PI3K/Akt signaling pathway in hepatocytes.** (**A-C**) Effects of NKAα1 inhibitor ouabain on the protein expressions of PI3K p110α subunit and p110β subunit protein expression as well as Akt phosphorylation in GlcN-incubated HepG2 cells. (**D-F**) Effects of NKAα1 knockout on the protein expressions of PI3K p110α subunit and p110β subunit protein expression as well as Akt phosphorylation in GlcN-incubated HepG2 cells. (**G-I**) Effects of NKAα1 activator DR-Ab on the protein expressions of PI3K p110α subunit and p110β subunit protein expression as well as Akt phosphorylation in GlcN-incubated HepG2 cells. (**J-L**) Effects of NKAα1 overexpression on the protein expressions of PI3K p110α subunit and p110β subunit protein expression as well as Akt phosphorylation in GlcN-incubated HepG2 cells. Data were expressed as Mean ± SEM. * P < 0.05 vs. Control (Con). † P < 0.05 vs. Vehicle (Veh) or wild-type (WT). The results were calculated from 4 to 6 independent experiments.

The PI3K/Akt signaling pathway is required for normal glucose metabolism, and its inactivation leads to insulin resistance through dysregulation of gluconeogenesis and glycogenesis 56. We also tested whether NKAα1 regulated hepatic glucose metabolism through the PI3K/Akt signaling pathway. It was found in the present study that the expressions of both p110α and p110β subunits of PI3K were downregulated in GlcN-challenged HepG2 cells. This is consistent with the previous reports 44. Inhibition of NKA with ouabain (**Figure 6A-C**) or genetic deletion of NKAα1 (**Figure 6D-F**) further decreased the expression of p110α subunit (**Figure 6A and 6D**) and phosphorylated Akt (**Figure 6C and 6F**), but had no significant effect on p110β subunit (**Figure 6B and 6E**) in GlcN-treated hepatocytes. Similar p110α subunit (**Figure S2F**) and phosphorylated Akt (**Figure S2G**) results were also confirmed in the primary cultured hepatocytes isolated from both NKAα1^+/+^ and NKAα1^+/-^ mice.

Notably, GlcN-induced downregulation of p110α subunit and phosphorylated Akt were reversed by either treatment with DR-Ab (**Figure 6G and 6I**) or NKAα1 overexpression (**Figure 6J and 6L**). However, neither of them affected the expression of p110β subunit (**Figure 6H and 6K**). Pharmacological manipulation of PI3K/Akt pathway by pretreatment with LY294002 (a PI3K inhibitor,**Figure S3**) or MK2206 (an Akt inhibitor, **Figure S4**) prevented the effect of DR-Ab on the protein expressions of PEPCK, G6pase, phosphorylated GSK3, phosphorylated GS, glucose content and as glycogen production in GlcN-treated HepG2 cells (**Figure S3-S4**). Similarly, NKAα1 overexpression also produced similar effects (**Figure S5-S6)**. Altogether, our results provided ample evidence that NKAα1 activation/overexpression improved hepatic insulin resistance via activating the PI3K/Akt signaling pathway.

### DR-Ab-mediated NKAα1 activation attenuated hyperglycemia and insulin resistance in obese diabetic mice

**Figure 7:**
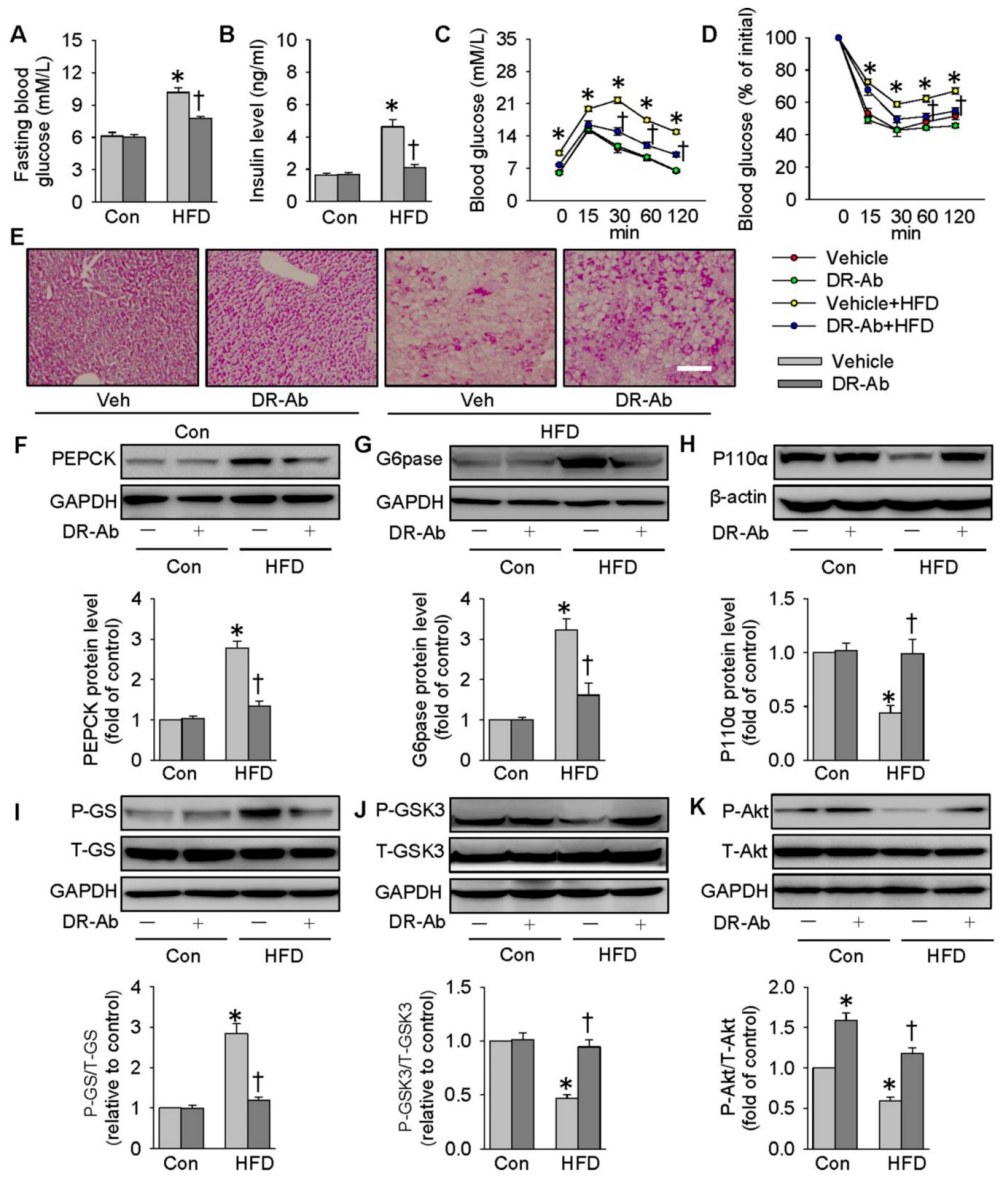
** DR-Ab-mediated NKAα1 activation attenuates hyperglycemia and insulin resistance in obese diabetic mice.** (**A**) Fasting blood glucose level, (**B**) serum insulin level, (**C**) GTT, (**D**) ITT, (**E**) Representative photographs of PAS staining in liver sections, showing that the decreased glycogen in HFD mice was prevented by DR-Ab treatment. Representative immunoblots and quantification analysis of PEPCK (**F**), G6pase (**G**), P110α (**H**), GS phosphorylation (**I**), GSK3 phosphorylation (**J**) and Akt phosphorylation (**K**) in the livers from mice. Data were expressed as Mean ± SEM. * P < 0.05 vs. Control (Con). † P < 0.05 vs. Vehicle or Vehicle+HFD. The data were calculated from 6 to 10 independent experiments.

We also created a mouse model of obese diabetes by feeding a HFD. As shown in** Figure 7**, feeding mice with HFD for 12 weeks significantly increased fasting blood glucose (**Figure 7A**) and serum insulin levels (**Figure 7B**). GTT and ITT results showed insulin resistance and glucose tolerance in these HFD mice (**Figure 7C-D**). HFD mice were intraperitoneally injected with DR-Ab (2 mg/kg/every other day) during the last 6 weeks. Immunofluoresenct staining showed the accumulation of DR-Ab in the liver after 6 weeks treatment (**Figure S7A**). More importantly, DR-Ab treatment reversed the above blood glucose and insulin changes (**Figure 7A-D**). PAS staining showed that the reduced glycogen store in HFD mice was significantly prevented by administration of DR-Ab (**Figure 7E**).

We also determined NKAα1 expression and activity in the livers of HFD mice. In line with the *in vitro* experiments, the membrane and total NKAα1 expressions as well as NKA activity were reduced in the liver tissues of HFD mice (**Figure S7B-E**). The reductions in membrane NKAα1 expression and NKA activity, but not total NKAα1 expression, were rescued by treatment with DR-Ab (**Figure S7B-E**). In summary, our data indicate that DR-Ab may be helpful to maintain normal function of NKAα1 through stabilizing plasma membrane NKAα1 in hepatic insulin resistance.

In parallel to the *in vitro* results, DR-Ab treatment strikingly attenuated the upregulations of PEPCK and G6pase protein expressions (**Figure 7F-G**), and the phosphorylation of GS in the liver tissues of HFD mice (**Figure 7I**). The protein expression levels of P110α (**Figure 7H**), GSK3 phosphorylation (**Figure 7J**), and Akt phosphorylation (**Figure 7K**) were reduced in obese diabetic livers, and these effects were also reversed by DR-Ab. As a result, our animal experiments confirmed that activation of NKAα1 by DR-Ab could ameliorate hepatic insulin resistance by regulation of gluconeogenesis and glycogen synthesis in obese diabetic mice.

### NKAα1 overexpression improved glucose metabolism and insulin resistance

**Figure 8:**
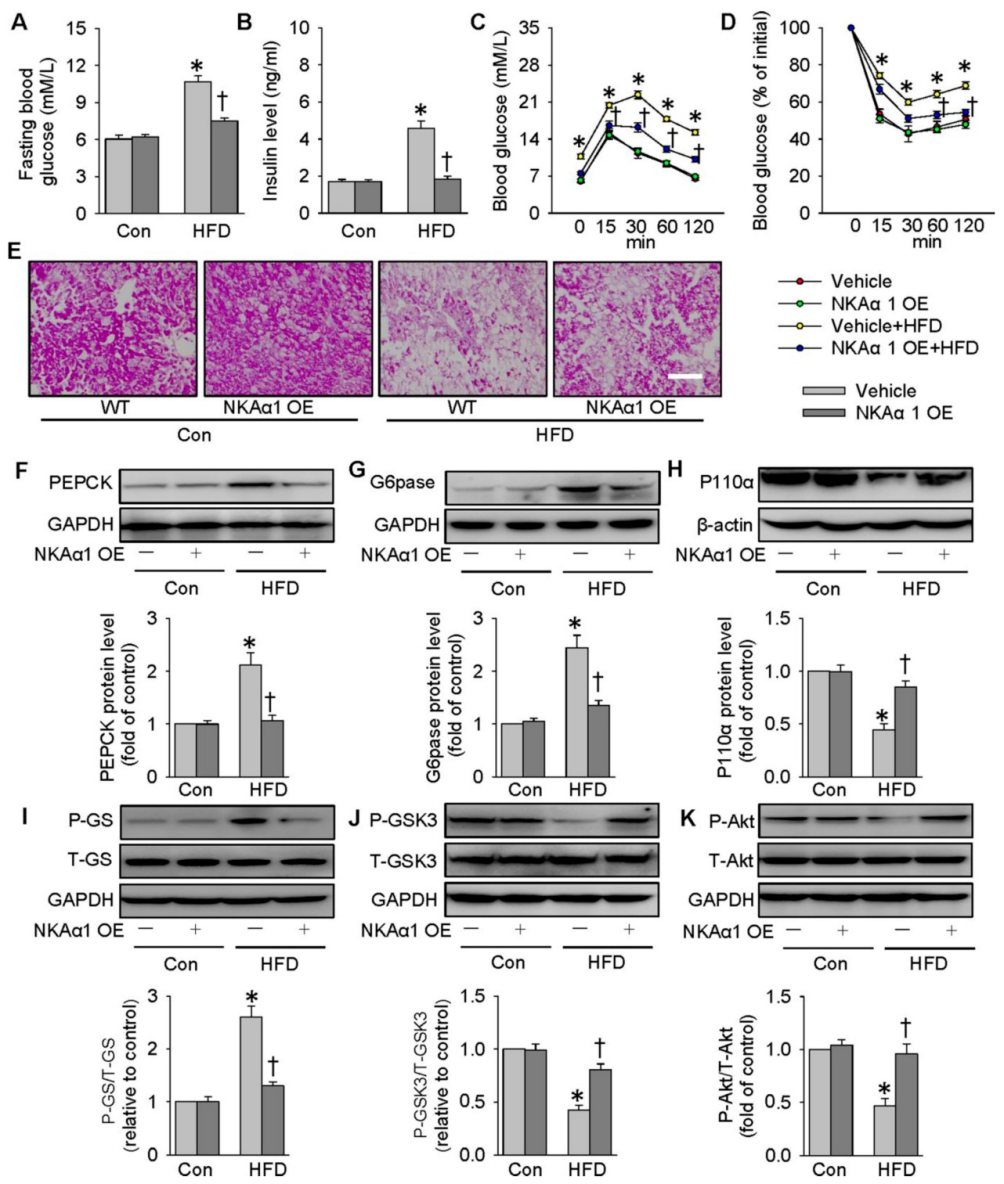
** NKAα1 overexpression improves glucose metabolism and insulin resistance.** (**A**) Fasting blood glucose level, (**B**) serum insulin level, (**C**) GTT, (**D**) ITT, (**E**) PAS staining in liver sections. Representative immunoblots and quantification analysis of PEPCK (**F**), G6pase (**G**), P110α (**H**), GS phosphorylation (**I**), GSK3 phosphorylation (**J**) and Akt phosphorylation (**K**) in the livers from mice. Data were expressed as Mean ± SEM. * P < 0.05 vs. Control (Con). † P < 0.05 vs. Vehicle or Vehicle+HFD. The results were calculated from 6 to 10 independent experiments.

Lentivirus-mediated NKAα1 overexpression led to high levels of NKAα1 in the liver tissues (**Figure S7F**). Delivery of NKAα1 lentiviral activation particles reduced both basal fasting blood glucose and serum insulin levels in HFD mice (**Figure 8A-B**). Furthermore, overexpression of NKAα1 improved glucose tolerance and ameliorated whole-body insulin resistance, as evidenced by GTT and ITT assay (**Figure 8C-D**). As shown by PAS staining results, the decreased glycogen levels in the livers from HFD mice were recovered by NKAα1 overexpression (**Figure 8E**). Consistent with the ability of DR-Ab to improve insulin sensitivity, liver overexpression of NKAα1 activated the PI3K/Akt signaling pathway to modulate the expressions of gluconeogenesis- and glycogenesis-related enzymes (**Figure 8F-K**), which was consistent with our *in vivo* data. In summary, NKAα1 is a strong suppressor of both hyperglycemia and insulin resistance in mice fed by a HFD.

### NKAα1 loss aggravated HFD-induced glucose metabolism disorders

**Figure 9:**
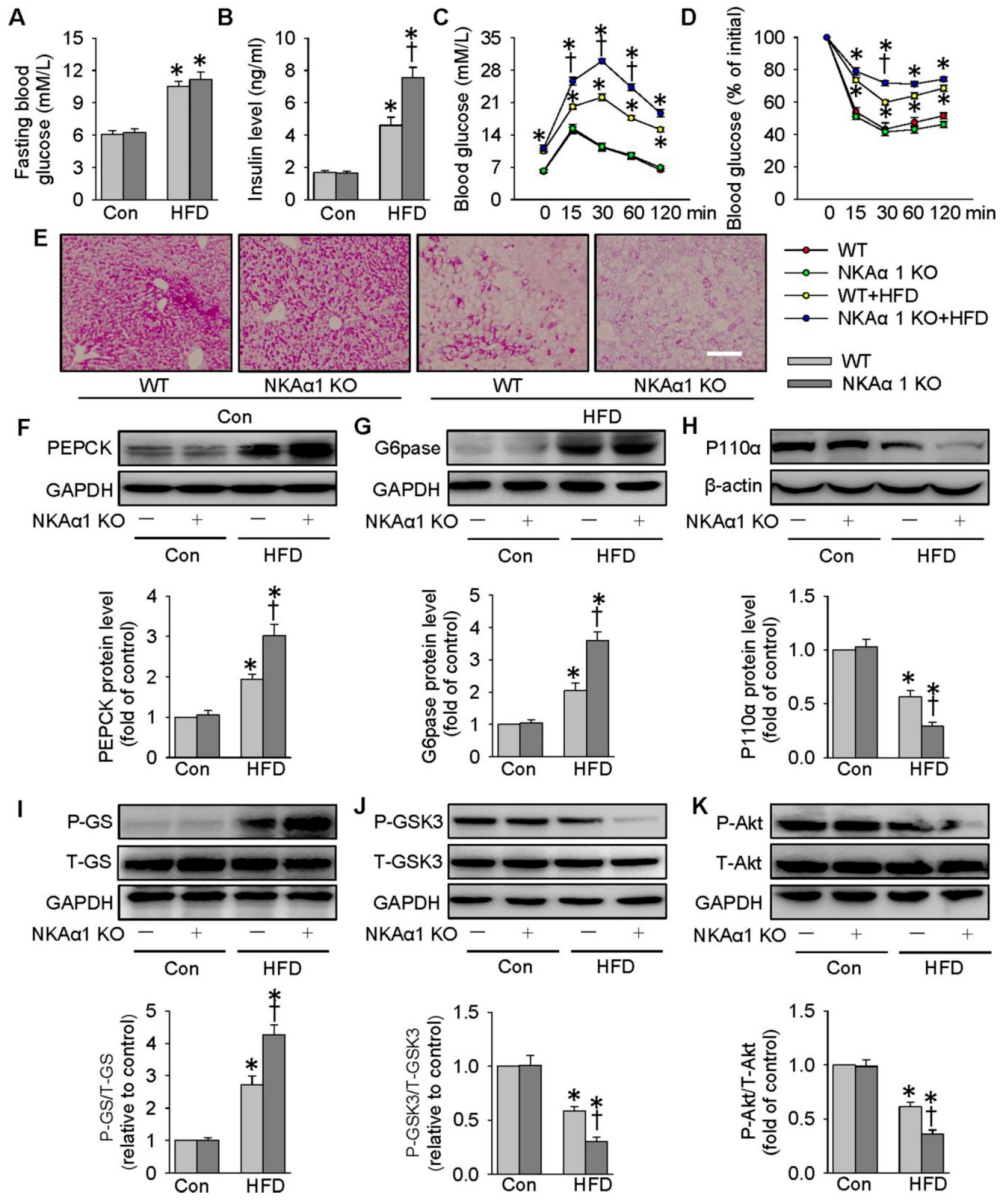
** NKAα1 knockout aggravated HFD-induced glucose metabolism disorders.** (**A**) Fasting blood glucose level, (**B**) serum insulin level, (**C**) GTT, (**D**) ITT, (**E**) PAS staining in liver sections. Representative immunoblots and quantification analysis of PEPCK (**F**), G6pase (**G**), P110α (**H**), GS phosphorylation (**I**), GSK3 phosphorylation (**J**) and Akt phosphorylation (**K**) in the livers from mice. Data were expressed as Mean ± SEM. * P < 0.05 vs. Control (Con). † P < 0.05 vs. wild-type (WT) or WT+HFD. The results were calculated from 6 to 10 independent experiments.

Eventually, the impact of NKAα1 gene knockout on hyperglycemia and insulin resistance in mice was determined. The heterozygous NKAα1^+/-^ mice displayed an obvious reduction in NKAα1 protein expression in the liver tissues, when compared to WT mice (**Figure S7G**). No significant difference was detected between NKAα1^+/+^ (WT) and NKAα1^+/-^ in terms of basal fasting blood glucose, serum insulin level, glucose tolerance or insulin sensitivity (**Figure 9**). Although there was no statistical difference in fasting blood glucose levels between HFD-fed WT mice and NKAα1^+/-^ mice **(Figure 9A)**, we observed a higher serum insulin level in HFD mice in NKAα1^+/-^ mice compared with WT (**Figure 9B**). GTT and ITT showed that mice with heterozygous global deficiency of NKAα1 exhibited exacerbated glucose intolerance and insulin resistance when compared with control mice (**Figure 9C-D**). NKAα1 deficiency mice also exhibited lower glycogen levels in the liver tissues of NKAα1^+/-^ HFD mice in comparison with WT HFD mice (**Figure 9E**). Accordingly, protein expressions of PEPCK (**Figure 9F**) and G6pase (**Figure 9G**) as well as phosphorylated GS (**Figure 9I**) were further elevated in HFD NKAα1^+/-^ mice-derived liver tissues. Conversely, the downregulated protein expressions of P110α (**Figure 9H**), phosphorylated GSK3 (**Figure 9J**), and phosphorylated Akt (**Figure 9K**) were also further dramatically reduced in the livers of HFD NKAα1^+/-^ mice.

## Discussion

As one of the classical ion pumps, the ion transport function of NKA in cells has been well studied 57. Over the last few decades, emerging evidence has recognized NKA as an important signal transducer for the development of new drugs 58. It has been reported that abnormal regulation of NKA activity is implicated in many metabolic disease, such as obesity, insulin resistance, and diabetes 59. Despite the advance in current knowledge of NKA in metabolic diseases, a clear definition of the role of NKA in obesity-related insulin resistance, especially in hepatic glucose metabolism, remains unsolved. In the present study, we showed that both NKA activity and NKAα1 expression were disrupted in HFD-induced livers and hepatocytes with insulin resistance. Importantly, we found that genetic downregulation of NKA activities in NKAα1^+/-^ mice led to aggravated hepatic glucose metabolism disorders and insulin resistance induced by HFD feeding. Conversely, upregulation of NKA function by overexpression of NKAα1 or targeting the DR region on NKA α1 with DR-Ab was shown to prevent hyperglycemia and insulin resistance in HFD mice. Mechanistically, activation of NKA function stimulated the PI3K/Akt signaling pathway to prevent gluconeogenesis and accelerate glycogen synthesis, thereby attenuating hyperglycemia and improving insulin resistance in mice induced by a HFD (**Figure S8**).

We first investigated whether NKA expression and function were altered in hepatic insulin resistance from both cell experiments and HFD mice. Our results revealed that NKAα1 protein and mRNA expressions were decreased in the liver tissues and hepatocytes during insulin resistance, suggesting that dysregulation of NKAα1 might be an adaptive response to hepatic insulin resistance. However, the underlying mechanisms by which HFD suppressed NKAα1 in the livers warrant further investigation. In addition, more research is required to examine the relationship between NKAα1 and pathological hepatic insulin resistance in clinical settings. Consistently, the plasma membrane expression of NKAα1 was also significantly declined in both GlcN-incubated hepatocytes and HFD mice-derived liver tissues. Thus, the downregulated total and membrane NKAα1 protein expressions may account for reductions in NKA activity and function in the process of hepatic insulin resistance. These findings drove us to investigate whether activation of NKA function by restoration of total and membrane NKAα1 protein ameliorated hyperglycemia and hepatic insulin resistance in HFD mice.

Our lab and other research groups have demonstrated that targeting the DR region of NKAα1 by DR-Ab can stabilize the membrane abundance of NKAα1 to grant neuroprotective effects 52, osteoporosis prevention 53, cardioprotection 32, 33, and renal protective effects 36. To study whether HFD-induced hepatic pathology and insulin resistance are related with impaired NKA function, we treated the HFD mice with DR-Ab which stimulates NKA activity. As expected, we found that DR-Ab treatment activated NKA function by protecting cell membrane NKAα1 from internalization, which helps to maintain normal NKA function in hepatocytes, therefore attenuating hyperglycemia and improving insulin resistance in obese diabetic mice. Likewise, activation of NKA function by hepatic upregulation of NKAα1 showed the similar therapeutic effects on hepatic insulin resistance in HFD mice. On the contrary, NKAα1^+/-^ mice with impaired NKA function exhibited the deteriorated hepatic insulin resistance challenged by a HFD. Collectively, reductions in NKA function rendered hepatocytes more vulnerable to glucose metabolism dysfunction in hepatic insulin resistance. Activation of NKA function by DR-Ab or NKAα1 overexpression ameliorated hyperglycemia and insulin resistance in obese diabetic mice. NKAα1 gene might be proposed as an effectively therapeutic target for glucose metabolic disorders and insulin resistance in the livers. Simultaneously, the DR region of NKA may be also a novel target for the evolution of drugs that stimulate NKA activity.

The imbalance in gluconeogenesis and glycogenesis is a critical event in the pathogenesis of metabolic syndrome, insulin resistance, and diabetes 60. In the present study, our results confirmed that inactivation of NKA function by its inhibitor ouabain or NKAα1 knockout promoted hepatic gluconeogenesis and glucose generation via upregulating the protein expressions and activities of both PEPCK and G6pase, whereas activation of NKA function by DR-Ab or NKAα1 overexpression had the opposite effects. In terms of glycogenesis, the reduced glycogen synthesis via regulation of GSK3-mediated GS activation in hepatocytes with insulin resistance, which is similar to the signal pathway of insulin in promoting glycogen synthesis 61, was aggravated by inhibited NKA function, but was attenuated by preserved NKA function. These above observations were further confirmed in primary NKAα1 knockout mice hepatocytes with insulin resistance. These results implied that the effective maintenance of normal NKA function prevented insulin resistance by accelerating glycogenesis and decreasing gluconeogenesis in the livers, thus ameliorating the imbalanced glucose homeostasis in obese diabetic mice.

The PI3K/Akt signaling pathway is a critical modulator in gluconeogenesis and glycogen synthesis during insulin resistance 62. Importantly, activation of NKA protects hearts from ischaemic injury in both cardiomyocytes and isolated hearts through activating the PI3K/Akt signaling pathway 63. These findings propel us to test whether the effects of NKA on gluconeogenesis and glycogen synthesis are associated with the PI3K/Akt signaling pathway. In this study, the decreased P110α of PI3K subunit and Akt phosphorylation levels in both hepatocytes with insulin resistance and obese diabetic livers were further worsened by NKA function destruction, but rescued by NKA function preservation. Of note, the effects of NKA function activation on gluconeogenesis and glycogenesis in GlcN-exposed HepG2 cells were prevented by pretreatment with the PI3K inhibitor LY294002 and the Akt inhibitor MK2206, respectively. Altogether, these results suggested that activation of NKA function reduced gluconeogenesis and increased glycogenesis via the PI3K/Akt signaling pathway in hepatocytes with insulin resistance and HFD mice. However, the molecular mechanisms of NKA in activating the PI3K/Akt signaling pathway remain unclear, which is worth further research.

## Conclusions

Both DR-Ab, an antibody that binds to the DR region of NKAα1 to activate NKA function, and NKAα1 overexpression were demonstrated to afford protection against HFD-induced hyperglycemia and hepatic insulin resistance by activating the PI3K/Akt signaling pathway. Hence, NKAα1 gene or the DR region of NKAα1 may function as potentially therapeutic targets to combat glucose metabolic disorders in obese diabetic mice. Moreover, the effects of NKAα1 on glucose uptake in muscle cells or adipocytes, glycogenolysis, and lipid metabolism have yet to be comprehensively understood in this study. These effects warrant in-depth research as they might contribute to the beneficial effects of NKAα1 activation or overexpression in the management of obese diabetes and insulin resistance.

## Supplementary Material

Supplementary figures and tables.

## Abbreviations

AGE: advanced glycation end-products
DMEM: dulbecco's modified eagle's medium
DR-Ab: DR region of NKAα1 subunit
FBS: fetal bovine serum
G6pase: glucose-6-phosphatase
GlcN: glucosamine
GS: glycogen synthase
GSK3: glycogen synthase kinase-3
GTT: glucose tolerance test
HFD: high-fat diet
ITT: insulin tolerance test
NEFA: non-esterified fatty acid
NKA: Na^+^/K^+^-ATPase
PEPCK: phosphorenolpyruvate carboxykinase
PI3K: phosphoinositide 3-kinase
