# Supplementary material for: DR-region of Na+/K+-ATPase is a target to ameliorate hepatic insulin resistance in obese diabetic mice
Source: Theranostics. 2020 May 15;10(14):6149–66. doi: 10.7150/thno.46053 (PMC7255017; doi:10.7150/thno.46053)
Supplement: Supplementary file 1 — Supplementary figures and tables. [file thnov10p6149s1.pdf]

Supplementary Data

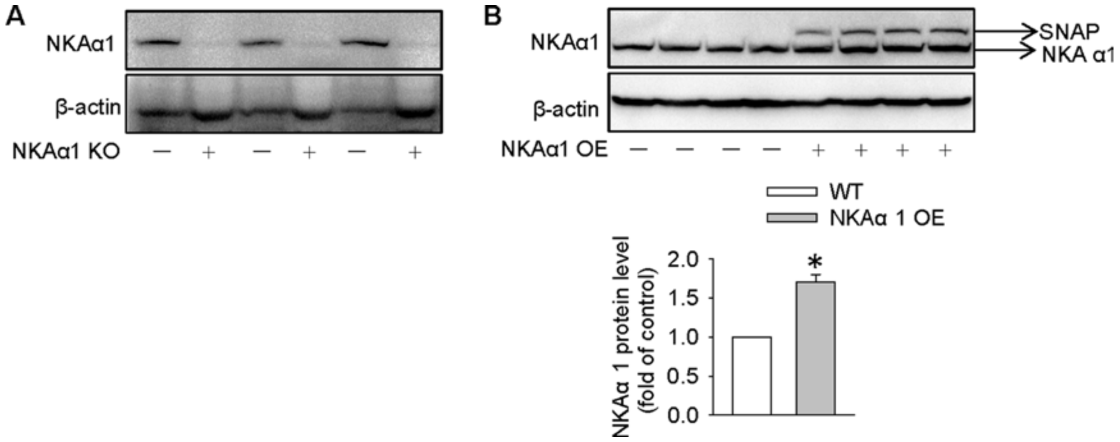

Figure S1. **The protein expression of NKAα1 in HepG2 cells**. (**A**) Effects of gene knockout on the protein expression of NKAα1 determined by western blot. (**B**) Effects of gene overexpression on the protein expression of NKAα1 determined by western blot. Data were expressed as Mean ± SEM. * $P < 0.05$ vs. wild-type (WT). The data were calculated from 4 to 8 independent experiments.

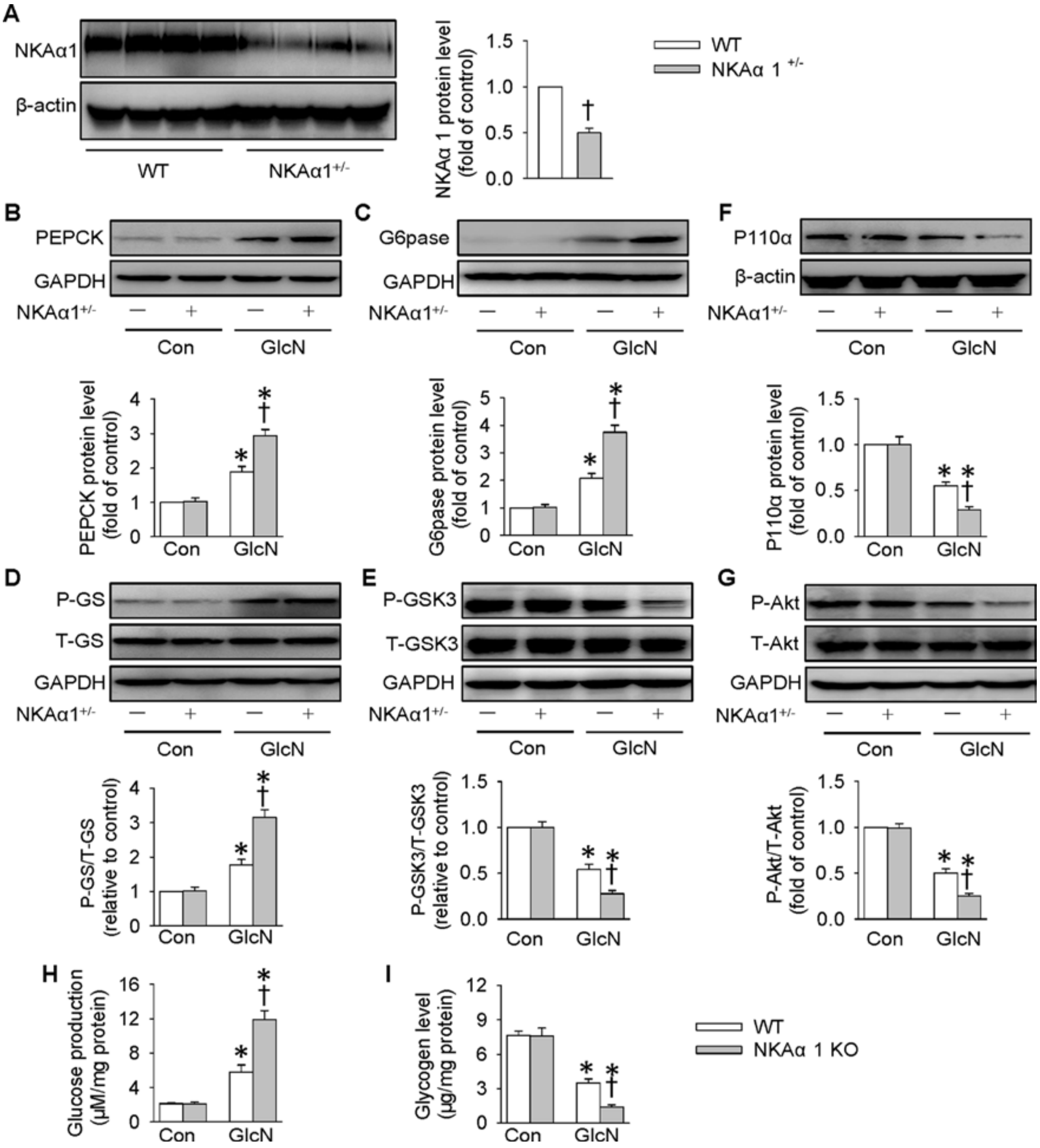

Figure S2. **Effects of NKAα1 knockout on gluconeogenesis and glycogenesis in GlcN-treated primary mouse hepatocytes.** (**A**) The protein expression of NKAα1 was examined in primary mouse hepatocytes from wild-type (WT) and NKAα1 knockout (NKAα1 KO) mice. Representative immunoblots and quantification analysis of PEPCK (**B**), G6pase (**C**), GS phosphorylation (**D**), GSK3 phosphorylation (**E**), P110α (**F**) and Akt phosphorylation (**G**). (**H**) Glucose production. (**I**) Glycogen levels. Data were expressed as Mean ± SEM. * $P < 0.05$ vs. Control (Con). † $P < 0.05$ vs. wild-type (WT). The data were calculated from 4 to 6 independent experiments.

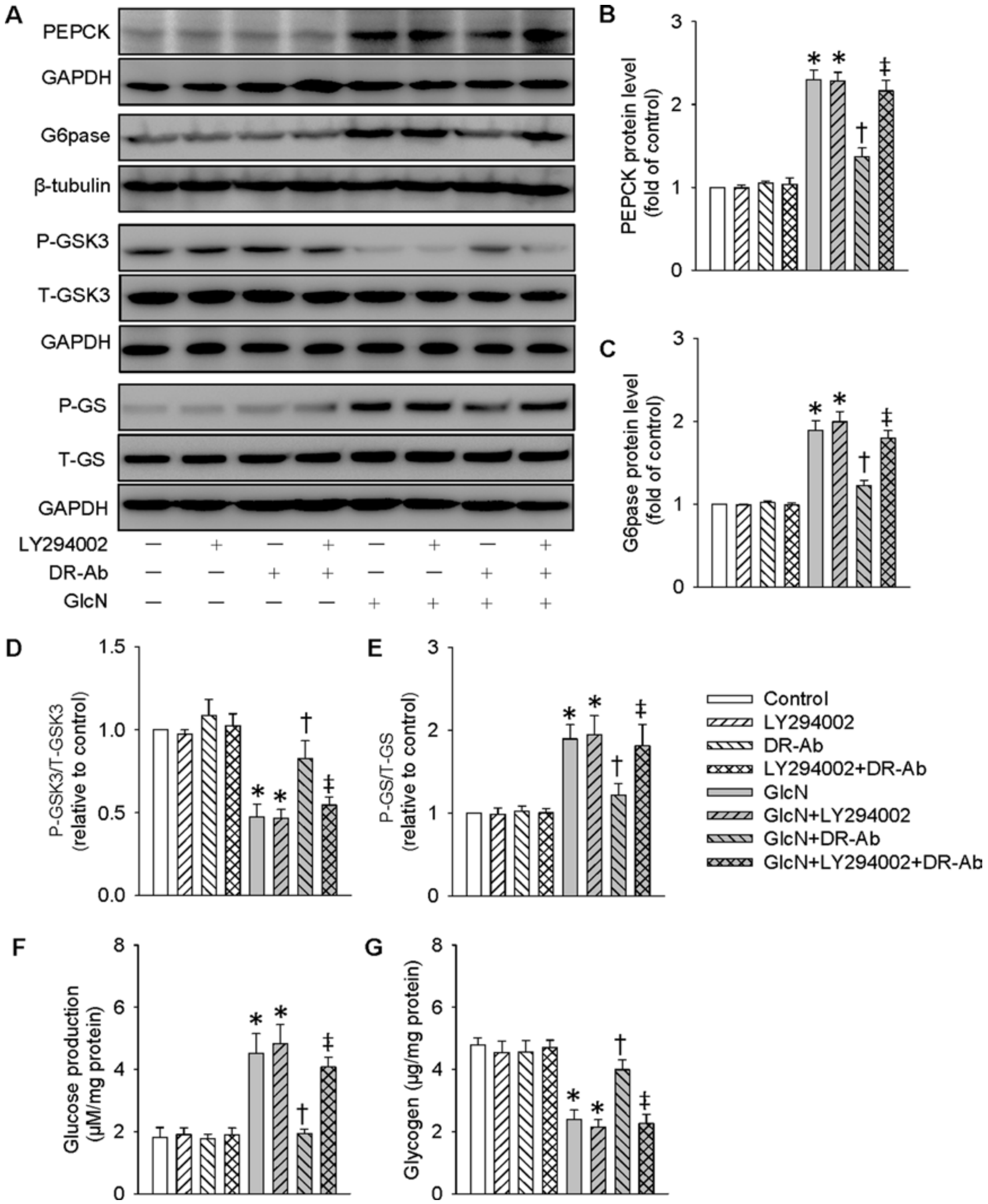

Figure S3. **PI3K inhibitor LY294002 prevented the effects of DR-Ab on gluconeogenesis and glycogenesis in GlcN-treated human HepG2 cells.** (**A**) Represented blots showing the protein expressions of PEPCK, G6pase, GSK3 phosphorylation and GS phosphorylation. The protein expression of PEPCK (**B**), G6pase (**C**), GSK3 phosphorylation (**D**) and GS phosphorylation (**E**) were quantitatively analyzed. (**F**) Glucose production. (**G**) Glycogen levels. Data were expressed as Mean ± SEM. * $P < 0.05$ vs. Control. † $P < 0.05$ vs. GlcN. ‡$P < 0.05$ vs. GlcN+DR-Ab. The data were calculated from 4 to 6 independent experiments.

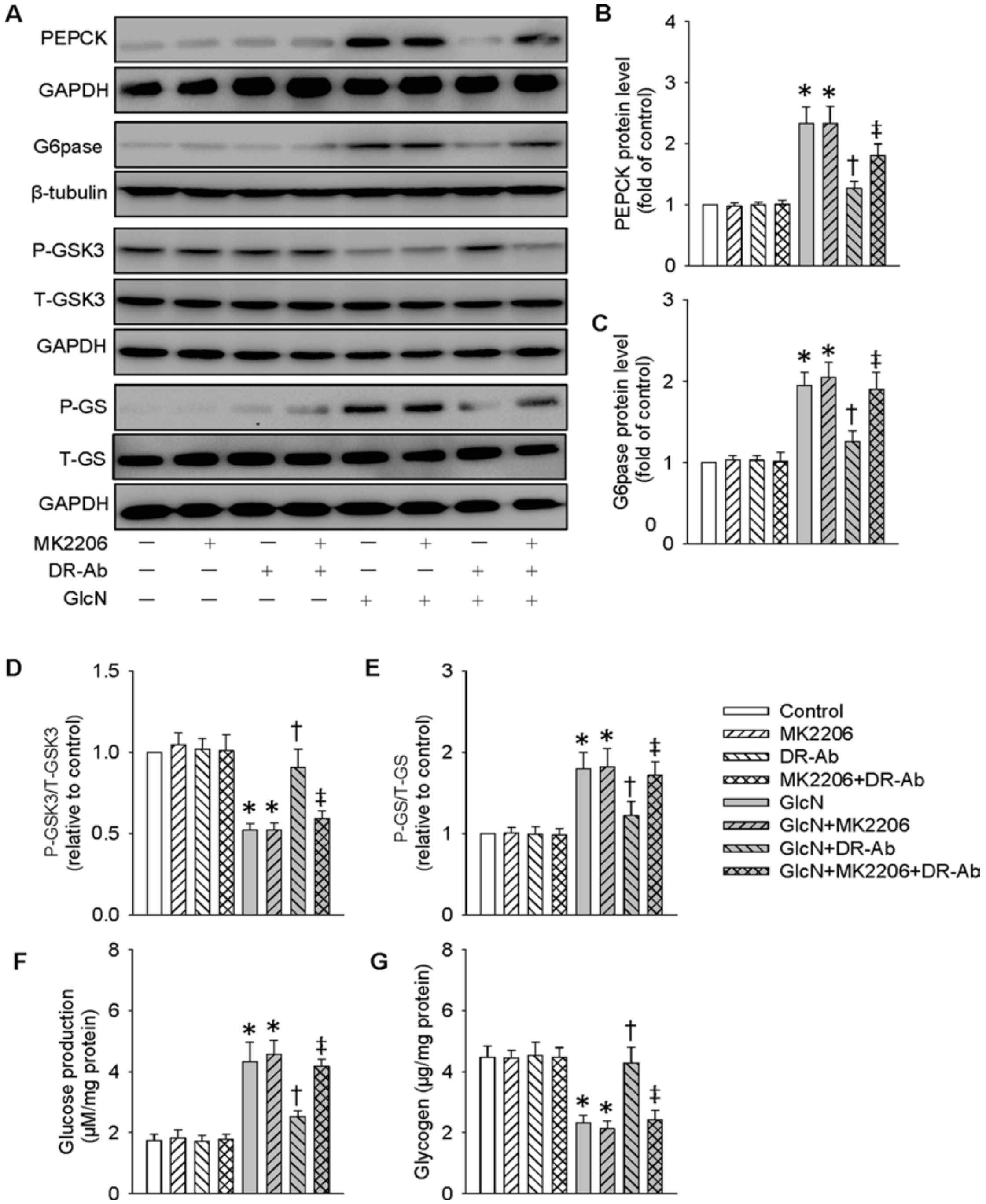

Figure S4. **Akt inhibitor MK2206 prevented the effects of DR-Ab on gluconeogenesis and glycogenesis in GlcN-treated human HepG2 cells.** (**A**) Represented blots showing the protein expressions of PEPCK, G6pase, GSK3 phosphorylation and GS phosphorylation. The protein expression of PEPCK (**B**), G6pase (**C**), GSK3 phosphorylation (**D**) and GS phosphorylation (**E**) were quantitatively analyzed. (**F**) Glucose production. (**G**) Glycogen levels. Data were expressed as Mean ± SEM. * $P < 0.05$ vs. Control. † $P < 0.05$ vs. GlcN. ‡$P < 0.05$ vs. GlcN+DR-Ab. The data were calculated from 4 to 6 independent experiments.

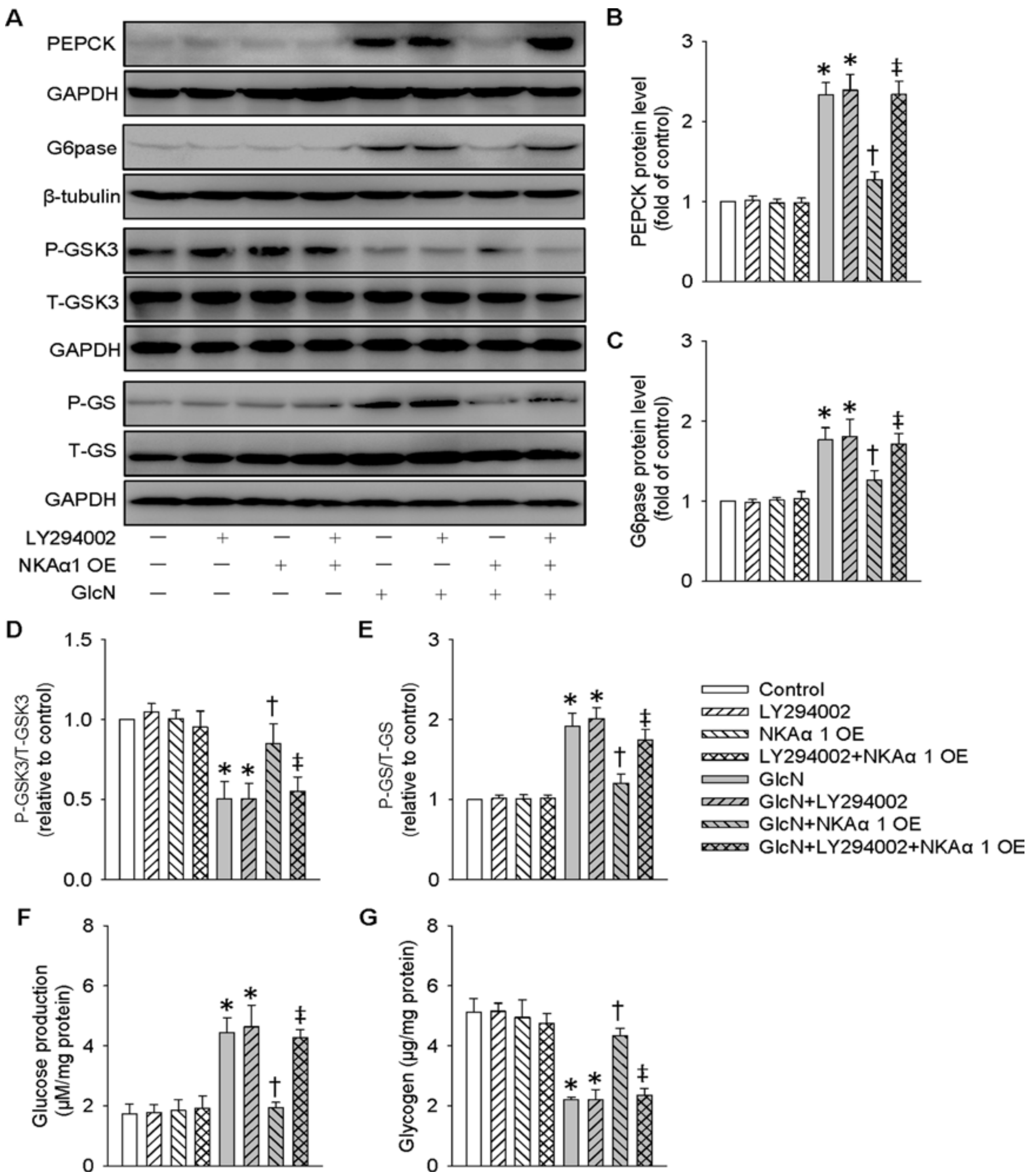

Figure S5. **PI3K inhibitor LY294002 prevented the effects of NKAα1 overexpression on gluconeogenesis and glycogenesis in GlcN-treated human HepG2 cells.** (**A**) Represented blots showing the protein expressions of PEPCK, G6pase, GSK3 phosphorylation and GS phosphorylation. The protein expression of PEPCK (**B**), G6pase (**C**), GSK3 phosphorylation (**D**) and GS phosphorylation (**E**) were quantitatively analyzed. (**F**) Glucose production. (**G**) Glycogen levels. Data were expressed as Mean ± SEM. * $P < 0.05$ vs. Control. † $P < 0.05$ vs. GlcN. ‡$P < 0.05$ vs. GlcN+NKAα1 OE. The data were calculated from 4 to 6 independent experiments.

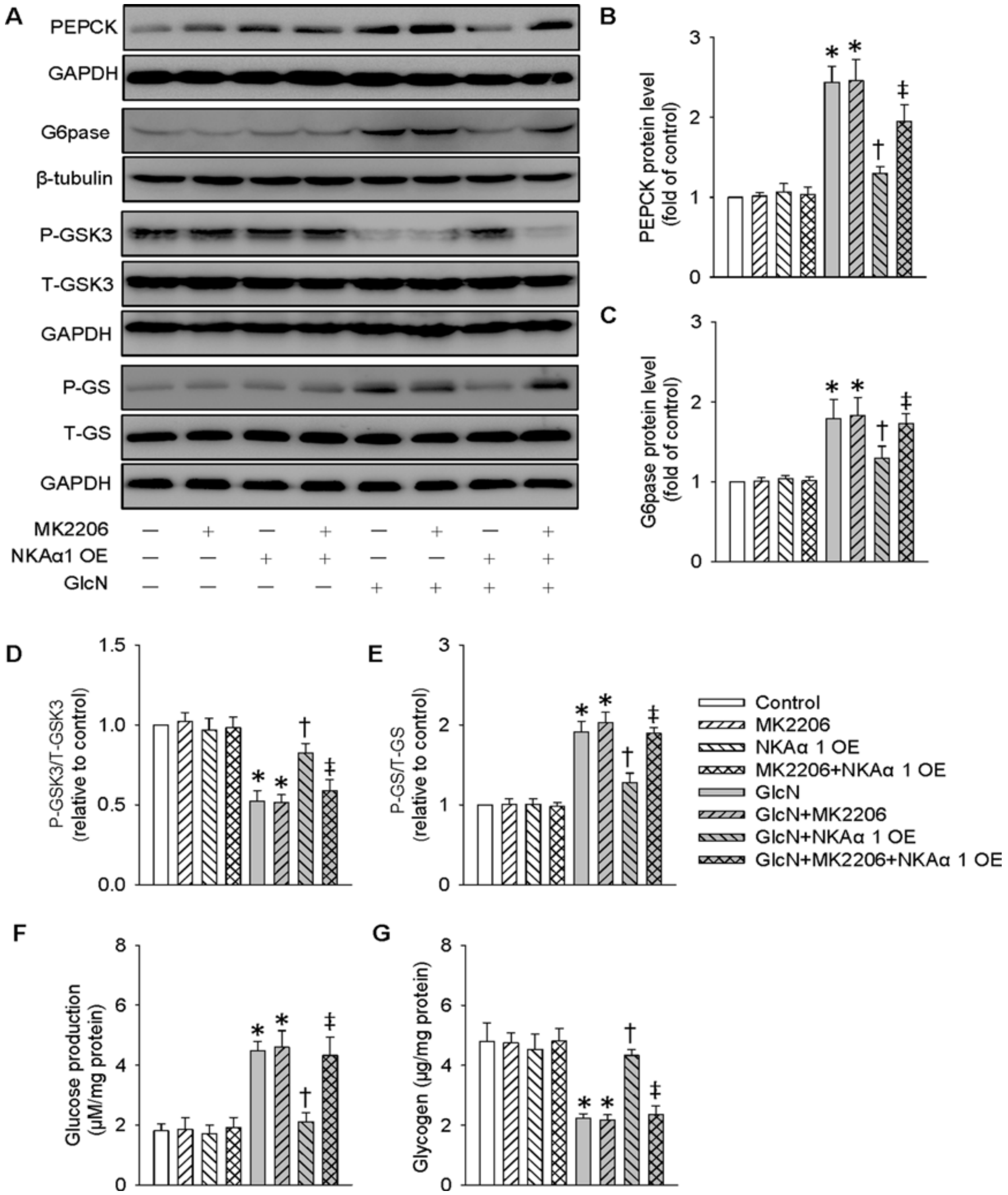

Figure S6. **Akt inhibitor MK2206 prevented the effects of NKAα1 overexpression on gluconeogenesis and glycogenesis in GlcN-treated human HepG2 cells.** (**A**) Represented blots showing the protein expressions of PEPCK, G6pase, GSK3 phosphorylation and GS phosphorylation. The protein expression of PEPCK (**B**), G6pase (**C**), GSK3 phosphorylation (**D**) and GS phosphorylation (**E**) were quantitatively analyzed. (**F**) Glucose production. (**G**) Glycogen levels. Data were expressed as Mean ± SEM. * $P < 0.05$ vs. Control. † $P < 0.05$ vs. GlcN. ‡$P < 0.05$ vs. GlcN+NKAα1 OE. The data were calculated from 4 to 6 independent experiments.

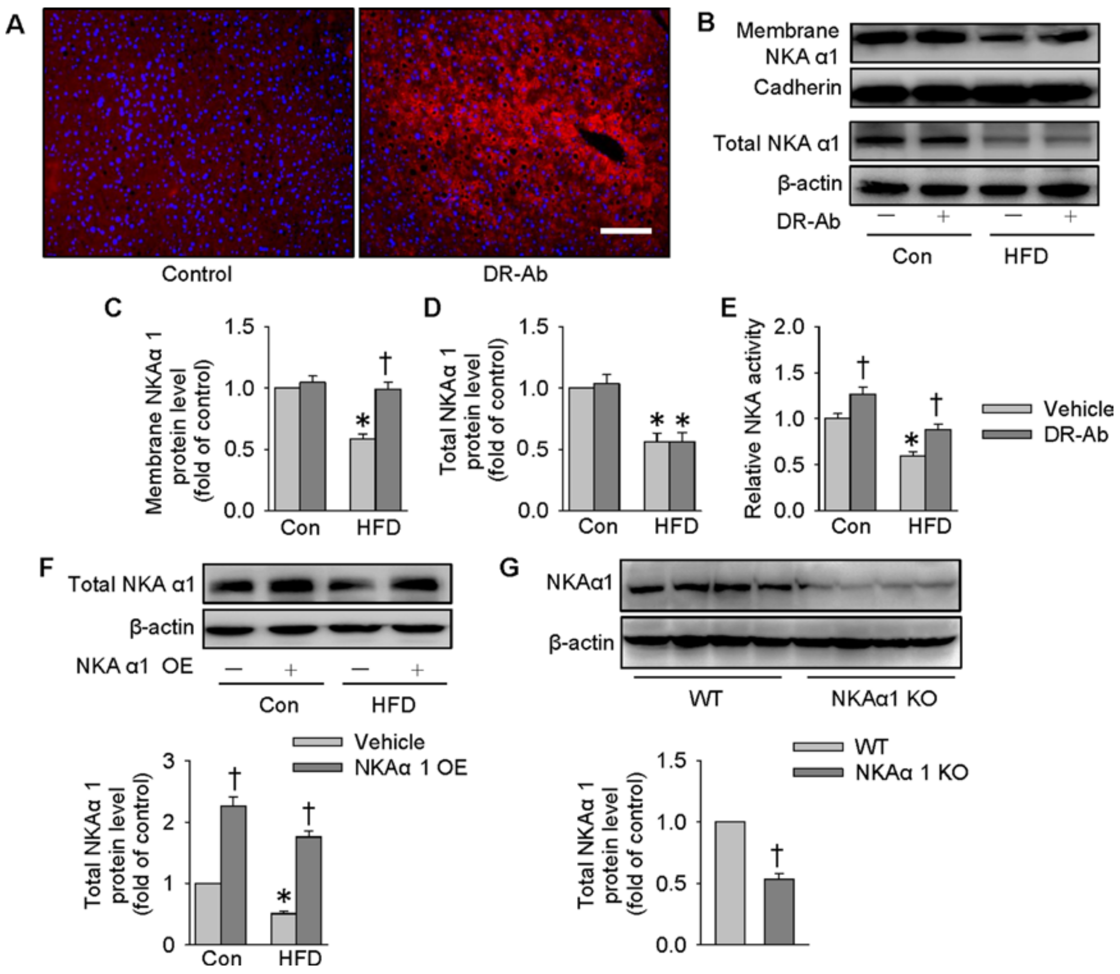

Figure S7. **The protein expression of NKAα1 in the livers from mice.** (**A**) The accumulation of DR-Ab in the liver was examined by immunofluorescence. (**B-D**) DR-Ab treatment reversed GlcN-induced loss of plasma membrane NKA α1, but had no effect on the total NKA α1 protein expression in the liver from HFD mice. (**E**) DR-Ab treatment reversed GlcN-induced downregulation of NKA activity in HFD mice. (**F**) Effects of gene overexpression of the protein expression of NKAα1 in the livers determined by western blot. (**G**) The protein expression of NKAα1 was examined in the livers from wild-type (WT) and NKAα1 knockout (NKAα1 KO) mice. Data were expressed as Mean ± SEM. * $P < 0.05$ vs. Control (Con), † $P < 0.05$ vs. wild-type (WT) or Vehicle. The data were calculated from 4 to 6 independent experiments.

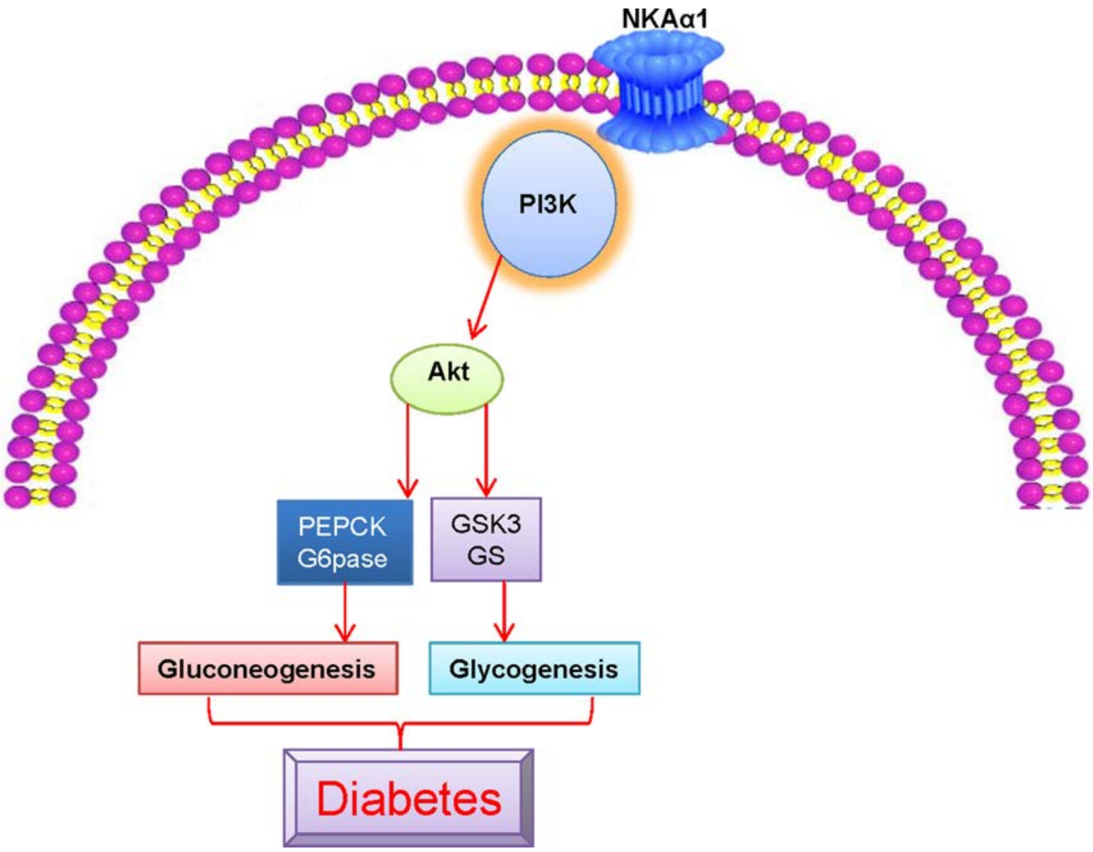

Figure S8. **Schematic illustration of the protective effects of NKAα1 against insulin resistance in diabetes**.

Table S1. Primers for Real-time quantitative PCR analysis in HepG2 cells.

	Primer	Sequence
NKAα1	Forward	5′-TCCATTATAATAGCCATCTT-3′
	Reverse	5′-AATGAAGCATGTAGCTCTA-3′
NKAα2	Forward	5′-AGCTGAACTTTCCCACGGAG-3′
	Reverse	5′-CTGATATGATGCCCACGCCT-3′
NKAα3	Forward	5′-TCAAGAAGGAGGTGGCTATG-3′
	Reverse	5′-GAGAAGCAGCCAGTGATGAT-3′
GAPDH	Forward	5'-CACTGCCACCCAGAAGA-3'
	Reverse	5'-GCTTCCCGTTCAGCTCA-3'

Table S2. Primers for Real-time quantitative PCR analysis in mice.

	Primer	Sequence
NKAα1	Forward	5′-GATCAGCATGGCCTATGGACAG-3′
	Reverse	5′-ACCGTTCTCAGCCAGAATCACA-3′
NKAα2	Forward	5′-GGCCGAAAATACCAAGTGGAT-3′
	Reverse	5′-TGTCCTGAGCTCGCTGATTG-3′
NKAα3	Forward	5′-TCAAGAAGGAGGTGGCTATG-3′
	Reverse	5′-GAGAAGCAGCCAGTGATGAT-3′
GAPDH	Forward	5'-AGGTTGTCTCCTGCGACTTCA-3'
	Reverse	5'-TGGTCCAGGGTT TCTTACTCC-3'
